# Olezarsen in Hypertriglyceridemia With High Cardiac Risk: A GRADE‐Assessed Meta‐Analysis of Randomized Trials With Trial Sequential Evidence

**DOI:** 10.1002/edm2.70220

**Published:** 2026-04-20

**Authors:** Ahmed Emara, Ameer Awashra, Heba Aboeldahab, Ahmed Farid Gadelmawla, Hamza A. Abdul‐Hafez, Abdullah M. Alharran, Amal A. Alsubaiei, Muneera Jasim AlRumaihi, Sara Ahmed Albuhmaid, Abdalhakim Shubietah

**Affiliations:** ^1^ Faculty of Medicine Al‐Azhar University Cairo Egypt; ^2^ Department of Medicine An‐Najah National University Nablus West Bank Palestine; ^3^ Clinical Research Department El‐Gomhoria General Hospital, MOHP Alexandria Egypt; ^4^ Faculty of Medicine Menoufia University Menoufia Egypt; ^5^ Arabian Gulf University Manama Kingdom of Bahrain; ^6^ Kuwait Institute for Medical Specializations Kuwait City Kuwait; ^7^ Department of Medicine Advocate Illinois Masonic Medical Center Chicago Illinois USA

**Keywords:** hypertriglyceridemia, meta‐analysis, olezarsen, randomized controlled trials

## Abstract

**Background:**

Hypertriglyceridemia is a widely prevalent disorder of lipid metabolism that increases the risk of cardiovascular disease and pancreatitis, and it often remains difficult to control even with standard treatments. Olezarsen, an antisense oligonucleotide that targets apolipoprotein C‐III (ApoC‐III), offers a new and promising option for lowering triglyceride levels.

**Methods:**

A systematic search of PubMed, Scopus, Web of Science, and Cochrane was conducted through September 2025 to identify randomized controlled trials (RCTs) comparing olezarsen with placebo in adults with hypertriglyceridemia at high cardiovascular risk. Dichotomous outcomes were analysed as risk ratios (RRs) and continuous outcomes as percentage mean differences (MDs), both with 95% confidence intervals (CIs).

**Results:**

Four RCTs (*n* = 1615 patients) were included. Olezarsen significantly reduced triglycerides (MD −47.71%, 95% CI −56.78 to −38.64, *p* < 0.0001), non‐HDL‐C (MD −22.11%, 95% CI −28.48 to −15.75, *p* < 0.0001), ApoC‐III (MD −68.93%, 95% CI −77.54 to −60.31, *p* < 0.0001), VLDL‐C (MD −48.52%, 95% CI −57.16 to −39.87, *p* < 0.0001), and ApoB (MD −10.67%, 95% CI −16.83 to −4.51, *p* = 0.0007), while increasing HDL‐C (MD 35.13%, 95% CI –27.30 to –42.96, *p* < 0.0001). LDL‐C showed no significant change. The risks of any or serious adverse events were comparable to placebo. Olezarsen was associated with fewer acute pancreatitis events (*p* = 0.035) but higher rates of liver enzyme elevations ≥ 3× ULN (*p* = 0.046).

**Conclusions:**

Olezarsen demonstrated consistent improvements in triglycerides and other atherogenic lipid parameters with an overall acceptable safety profile. These findings suggest that olezarsen may be a useful adjunct option for patients with persistent hypertriglyceridemia despite standard therapy. Further large‐scale and long‐term studies are needed to confirm its cardiovascular and safety outcomes.

## Graphical abstract

This meta‐analysis of randomized trials evaluates olezarsen for hypertriglyceridemia in patients at high cardiovascular risk. Olezarsen significantly improved triglycerides and other atherogenic lipid parameters with generally acceptable safety, supporting its potential as a promising adjunct therapy while highlighting the need for larger and longer‐term outcome trials.

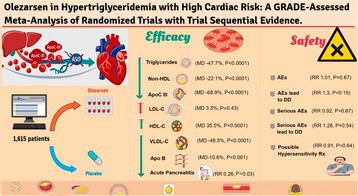

**Abbreviations**
AEs: adverse events
ALT: alanine aminotransferase
ApoB: apolipoprotein B
ApoC‐III: apolipoprotein C‐III
AST: aspartate aminotransferase
BMI: body mass index
CI: confidence interval
CKD: chronic kidney disease
eGFR: estimated glomerular filtration rate
Hb: haemoglobin
HDL‐C: high density lipoprotein cholesterol
LDL‐C: low density lipoprotein cholesterol
MD: mean difference
PCSK9: proprotein convertase subtilisin/kexin type 9
RCT: randomized controlled trial
ROB: risk of bias
RR: risk ratio
SAEs: serious adverse events
siRNA: small interfering RNA
TG: triglycerides
TSA: trial sequential analysis
ULN: upper limit of normal
VLDL‐C: very low‐density lipoprotein cholesterol

## 1 Introduction

Hypertriglyceridemia is a prevalent lipid disorder characterized by elevated levels of triglycerides (TG). It is typically classified as mild (150–399 mg/dL), moderate (400–999 mg/dL), or severe (≥ 1000 mg/dL) hypertriglyceridemia [1, 2]. It is a well‐recognized, independent risk factor for atherosclerotic cardiovascular diseases and pancreatitis [2, 3]. Despite widespread use of lifestyle measures and standard lipid‐lowering therapies, including fibrates, niacin, omega‐3 fatty acids, and statins, many retain a significant residual cardiovascular risk, leaving a clear unmet need for more effective, targeted treatments [4, 5].

Apolipoprotein C‐III (ApoC‐III) plays a central role in TG metabolism by inhibiting lipoprotein lipase and slowing hepatic clearance of TG‐rich lipoproteins, therapy promoting hypertriglyceridemia [6, 7]. Olezarsen is an investigational N‐acetylgalactosamine conjugated antisense oligonucleotide that has emerged as a novel therapeutic agent to inhibit the production of ApoC‐III in the liver by targeting the messenger RNA (mRNA) [6, 8, 9], resulting in marked reductions in circulating ApoC‐III protein. By lowering ApoC‐III, olezarsen accelerates lipolysis and enhances clearance of TG‐rich particles, addressing a key mechanistic driver of elevated TG levels [6, 7, 9]. In clinical trials, it has been administered subcutaneously at 50 mg or 80 mg once monthly. It demonstrates rapid absorption, predominant hepatic distribution, and a terminal half‐life of approximately 4 weeks, supporting sustained pharmacodynamic effects and monthly dosing.

Recent studies and clinical trials have reported olezarsen's ability to significantly reduce TG, ApoC‐III, and atherogenic lipoprotein levels, while generally showing a tolerable safety profile in populations at elevated cardiovascular risk [4, 8]. Treatment with olezarsen consistently reduced TG and other atherogenic markers such as VLDL‐C, non‐HDL‐C, and apolipoprotein B (ApoB), while generally producing favourable or neutral effects on LDL‐C and HDL‐C [4, 8, 10, 11]. Safety data have been reassuring to date, with no consistent signal of severe thrombocytopenia or clinically significant hepatic or renal toxicity [4, 8, 11].

While individual controlled trials and some systematic reviews have established the efficacy and safety of olezarsen [4, 8, 10], the rapid emergence of new data from large phase three trials necessitates an updated and comprehensive synthesis of the evidence.

Given these promising mechanistic and clinical findings, we performed a systematic review and meta‐analysis of randomized trials evaluating olezarsen in adults with hypertriglyceridemia at high cardiac risk. Our objective was to quantify olezarsen's effects on the lipid profile and to synthesize the evidence on safety. To strengthen inference and assess the significance of the results, we complemented the meta‐analysis with trial sequential analysis (TSA), GRADE certainty assessments, and predefined subgroup analyses based on follow‐ups and doses of olezarsen.

## 2 Methods

### 2.1 Protocol Registration

We conducted this systematic review and meta‐analysis following the PRISMA 2020 statement [12] (Table S1) and the Cochrane Handbook of Interventions [13]. The review protocol was prospectively registered in PROSPERO under the ID: CRD420251178073.

### 2.2 Data Sources and Search Strategy

We systematically searched four electronic databases: PubMed, Cochrane CENTRAL, Scopus, and Web of Science, from inception to September 21, 2025. The search included both free‐text terms and controlled vocabulary (MeSH/Emtree) for “Olezarsen”, Hypertriglyceridemia”, and “Randomized Controlled Trial” as shown in Table S2. No restrictions were placed on the language, publication date, or geographic location.

### 2.3 Eligibility Criteria

Randomized controlled trials (RCTs) that were published in English peer‐reviewed journals and met the following PICO framework:

- Population (P): Adults ≥ 18 years old with hypertriglyceridemia (defined as fasting triglycerides > 150 mg/dL) with high cardiac risk (defined by elevated ASCVD risk score or risk factors such as diabetes, hypertension, dyslipidemia, or smoking), on stable background lipid‐lowering therapy, which varied across studies but primarily included stable statin therapy and/or other maximally tolerated lipid‐lowering therapies (such as omega‐3 fatty acids, fibrates, ezetimibe, or PCSK9 inhibitors). We included phase 2 and 3 trials that evaluated participants with acquired hypertriglyceridemia or familial disorders such as familial chylomicronemia syndrome (FCS) that cause hypertriglyceridemia.
- Intervention (I): Olezarsen, administered subcutaneously once monthly (across 50 and 80 mg doses), as an adjunct to the patient's background lipid‐lowering therapy.
- Comparator (C): Matching placebo, administered subcutaneously once monthly, as an adjunct to the patient's background lipid‐lowering therapy.
- Outcomes (O): *Primary efficacy outcomes*: Mean percentage change in triglycerides level from the baseline and achievement of triglycerides level < 150 mg/dL. *Secondary outcomes*: *efficacy outcomes*: Mean percentage changes in lipid parameters such as LDL‐C, non‐HDL‐C, VLDL‐C, HDL‐C, ApoB, and ApoC‐III, and remnant cholesterol, while the safety outcomes included any adverse events (AEs), AEs leading to drug discontinuation, serious AEs, serious AEs leading to drug discontinuation, possible hypersensitivity reaction, injection site reaction, acute pancreatitis, all abnormal laboratory findings, decrease in eGFR ≥ 30%, urinary protein: creatinine ratio ≥ 1000, ALT or AST level ≥ 5× ULN, total bilirubin level ≥ 2× ULN, ALT or AST level ≥ 3× ULN, and change in HbA1c from baseline. All outcomes were calculated at the last follow‐up period.

The exclusion criteria included animal studies, narrative reviews, books, theses, non‐randomized or quasi‐experimental studies, observational designs (cohort or case–control), incomplete data, conference abstracts, protocols, letters, case reports, and case series.

### 2.4 Study Selection

Search results were imported into EndNote (version 20) for deduplication. Two reviewers independently (A.M.A. and A.A.A) screened titles and abstracts using Rayyan online software [14], followed by full‐text assessment for final eligibility. Discrepancies were resolved by adjudication with a third reviewer (A.E.).

### 2.5 Data Extraction

Three reviewers independently (A.E., A.F.G., and A.A.A.) extracted the data using a standardized spreadsheet. The extracted data included the following.

- *Study characteristics*: (study ID, trial name, design, phase, blinding, register number, location, number of centres, study period, sample size, study arms, inclusion criteria, primary outcomes, and follow‐up duration).
- *Baseline patient variables*: (total group participants, age, sex (male), BMI, diabetes, ASCVD, history of pancreatitis, chronic kidney disease (CKD), triglycerides level, LDL‐C, non‐HDL‐C, HbA1‐C, VLDL‐C, ApoC‐III, ApoB, and medications (statins, ezetimibe, fibrates, and omega‐3 fatty acids)).
- *Outcomes*: all predefined efficacy and safety endpoints as mentioned before

### 2.6 Quality Assessment

Two authors independently (A.A.A. and M.J.A.) assessed the methodological quality of the included RCTs using the Cochrane Risk of Bias 2 (ROB2) tool [15]. This evaluation covered five key domains: randomization, adherence to the assigned intervention, handling of missing outcome data, measurement of outcomes, and selective reporting. Each domain was rated as “low risk,” “some concerns,” or “high risk,” leading to an overall judgement. Any disagreements were resolved by consulting a third author (A.S.).

The certainty of evidence was evaluated using the GRADE approach by H.A., considering the risk of bias, inconsistency, indirectness, imprecision, and others (publication bias) [16].

### 2.7 Statistical Analysis

#### 2.7.1 Pairwise Meta‐Analysis

All statistical analyses were performed using R software (version 4.4.2) with the “meta” package [17]. Continuous outcomes were summarized as mean differences (MDs) with corresponding 95% confidence intervals (CIs), whereas dichotomous outcomes were expressed as risk ratios (RRs) with 95% CIs. For each study, the percentage change in lipid parameters was calculated or extracted as:

$$\begin{array}{c} \text{Percentage Change}\;\left(\%\right)\\ =\frac{\left(\text{Mean Value}\;\mathrm{at}\;\text{Follow}-\mathrm{up}-\text{Mean Baseline Value}\right)}{\text{Mean Baseline Value}}\times 100 \end{array}$$

When studies did not directly report percentage change, it was derived from available baseline and follow‐up means. The corresponding standard deviations (SDs) of percentage change were calculated or estimated from reported standard errors, confidence intervals, or *p*‐values following Cochrane Handbook guidance. Statistical significance was defined as a *p* < 0.05. For trials with zero‐event cells, a continuity correction of 0.5 was used to enable effect size calculation.

The random effect model was used in all measured outcomes as it accommodates a more significant standard error in the pooled estimate, making it suitable for inconsistent or controversial estimates. Assessment of publication bias, such as with Egger's regression test, was not performed due to the limited number of included studies (*n* = 4) as the test lacks sufficient power with fewer than 10 studies.

#### 2.7.2 Heterogeneity

Between‐study heterogeneity was evaluated using the *I* ^2^ statistic and the chi‐square (*χ* ^2^) test. The *I* ^2^ values were interpreted according to the Cochrane Handbook [13] as follows: 0%–30% (not important), 30%–50% (moderate), 50%–75% (substantial), and 75%–100% (considerable). A *p* < 0.1 from the *χ* ^2^ test was considered indicative of statistically significant heterogeneity.

#### 2.7.3 Sensitivity Analysis and Subgrouping

Sensitivity analyses were conducted using a leave‐one‐out approach to explore the robustness of the pooled estimates and potential sources of heterogeneity. Additionally, subgroup analyses were performed according to the administered olezarsen doses (50 and 80 mg) and follow‐up period (6 and 12 months).

#### 2.7.4 Trial Sequential Analysis

To evaluate the conclusiveness of cumulative evidence and to control for potential type I errors due to repeated significance testing, we conducted TSA using the TSA software (version 0.9.5.10 Beta) [18, 19]. The required information size (RIS) was calculated assuming a two‐sided alpha of 0.05% and 80% power. The TSA analysis provides a conclusive threshold for estimating the efficacy of olezarsen drug using a random‐effect model with 95% CI and diversity‐adjusted RIS to accommodate heterogeneity between studies. O'Brien–Fleming monitoring boundaries were performed to define thresholds for the true estimate from random error or futility. When the cumulative *Z*‐curve crossed the O'Brien–Fleming monitoring boundary or entered the futility area, it indicated that the evidence was sufficient to accept or reject the anticipated intervention effect, with no further studies required. If the *Z*‐curve did not cross any boundaries and the RIS was not reached, the evidence was considered inconclusive, indicating the need for further studies.

## 3 Results

### 3.1 Search Results

**FIGURE 1:**
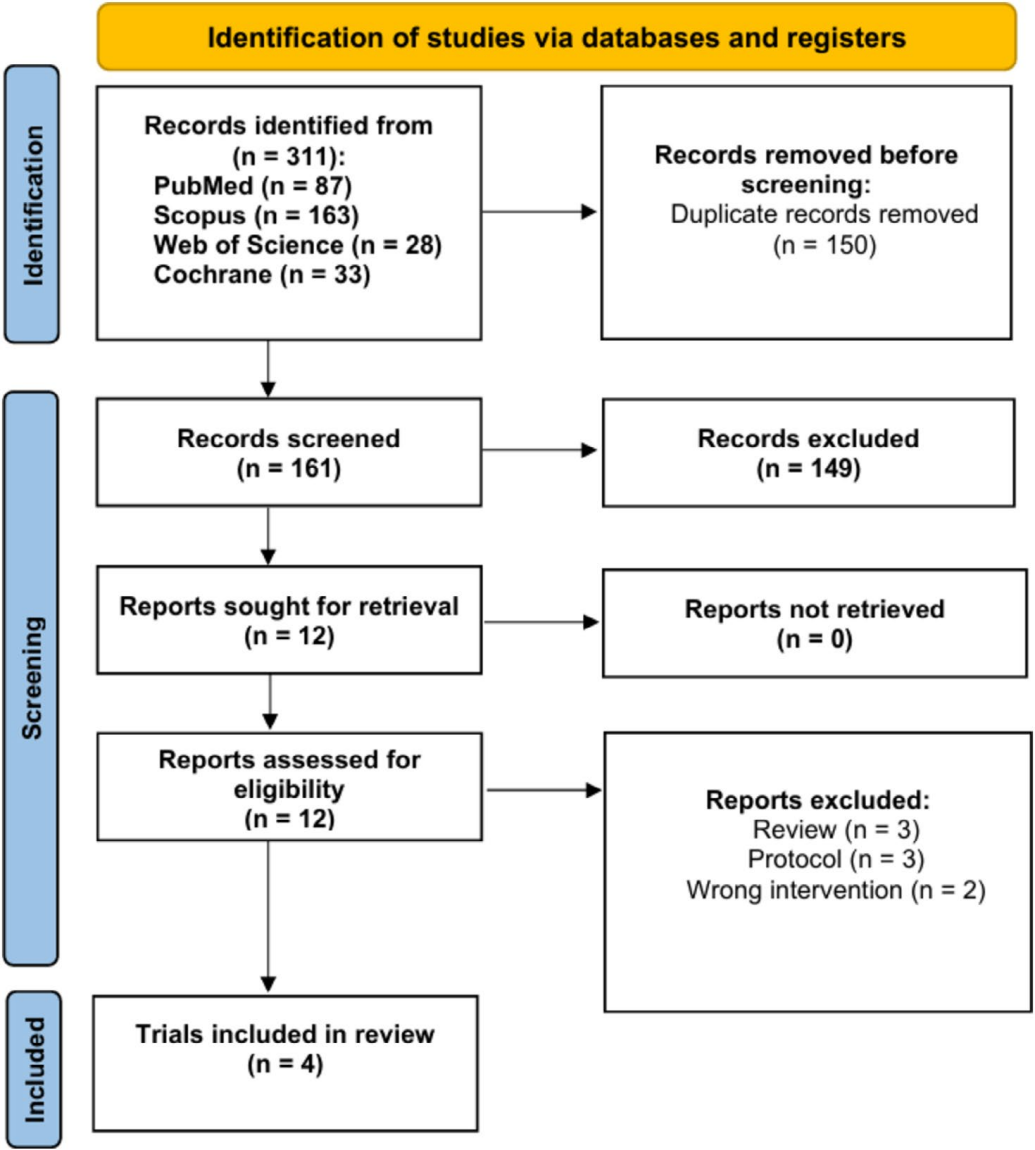
PRISMA flow chart for the systematic search and selection process.

The initial literature search yielded 311 records. By removing 150 duplicate records using EndNote (Clarivate Analytics, USA), we narrowed the dataset to 161 records for screening. We excluded 149 ineligible records based on the title and abstract information. Through a comprehensive full‐text review, we identified four trials that met the inclusion criteria for our review [8, 20, 21, 22]. No further papers were included after manually searching the references of the included studies. The PRISMA flow diagram visually illustrates our study selection process, outlining the steps to identify suitable research (Figure 1).

### 3.2 Characteristics of the Included Studies

Our study encompassed four RCTs [8, 20, 21, 22] offering a comprehensive evaluation of the safety and efficacy of olezarsen in patients with hypertriglyceridemia with high cardiac risk. The total number of patients in our study was 1615, with 1200 patients in the olezarsen group and 415 in the placebo group. Across the included trials, the geographic scope of the studies was extensive, encompassing countries worldwide, including North America (United States and Canada) and Europe. All included trials reported a multicentre design, involving participants recruited from more than one clinical site and included both phases 2 and 3. The follow‐up period ranged from 6 months up to 12 months. All included trials evaluated the 50 and 80 mg doses; however, the study by Tardif et al., 2022 investigated only the 50 mg dose. The mean age of participants was 62.38 ± 10.98 years, and the cohort was predominantly male (59.31%), with female patients comprising 40.6%. The baseline triglyceride level was 344.51 ± 547.69 mg/dL, whereas the baseline ApoC‐III level was 15.99 ± 5.39 mg/dL. More details about the summary of included studies and baseline characteristics of patients were presented in Tables 1 and 2.

**TABLE 1:** Summary of included trials.

Study ID	Trial name	Study design	Phase	Blinding	Register no.	Location	No of centres	Study period	Total sample size	Study arms		Inclusion criteria			Primary outcomes	Follow up, mo
										Intervention (s)	Comparator	Age	Condition	Fasting triglycerides		
Bergmark et al. 2025	Essence–TIMI 73b	RCT	Phase 3	Double‐blind	NCT05610280	North America and Europe	160	November 2022–February 2024	1349	Olezarsen (50 mg or 80 mg), monthly subcutaneous	Placebo	≥ 18	Moderate hypertriglyceridemia plus an increased risk of atherosclerotic cardiovascular disease or severe hypertriglyceridemia	Moderate hypertriglyceridemia (triglyceride level, 150–499 mg per deciliter [1.7–5.6 mmol per litre]). Severe hypertriglyceridemia (triglyceride level, ≥ 500 mg per deciliter [≥ 5.6 mmol per litre])	Change in triglyceride level	6,12
Bergmark et al. 2024	Bridge–TIMI 73a	RCT	Phase 2b	Double‐blind	NCT05355402	North America (United States and Canada)	24	June–September 2022	154	Olezarsen (50 mg or 80 mg), monthly subcutaneous	Placebo	≥ 18	Moderate hypertriglyceridemia plus an increased risk of atherosclerotic cardiovascular disease or severe hypertriglyceridemia	Fasting triglyceride levels of 41.5 mg per deciliter (2.7 mmol per litre; interquartile range, 192–324 mg per deciliter [2.2–3.7 mmol per litre])	Change in triglyceride level	6,12
Stroes et al. 2024	Balance	RCT	Phase 3	Double‐blind	NCT04568434	11 countries	29	November 2020–October 2023	66	Olezarsen (50 mg or 80 mg), monthly subcutaneous	Placebo	≥ 18	Severe hypertriglyceridemia and suspected familial chylomicronemia syndrome	Fasting triglyceride levels of 880 mg per deciliter (9.9 mmol per litre) or higher	Change in triglyceride level	6,12
Tardif et al. 2022	Olezarsen Study	RCT	Phase 2	Double‐blind	NCT03385239	North America (United States and Canada)	41	February 2018–May 2019	46	Olezarsen 50 mg, monthly subcutaneous	Placebo	≥ 18	Triglyceride levels 200–500 mg/dL (2.26–5.65 mmol/L) and at high risk for or with established ASCVD	Fasting triglyceride level was 262 (222–329) mg/dL [2.96 (2.51–3.71) mmol/L]	Change in triglyceride level	6,12

Abbreviations: ASCVD, atherosclerotic cardiovascular disease; Mo, months; RCT, randomized controlled trial.

**TABLE 2:** Baseline characteristics of included studies.

Trial name	Groups	Total participants	Age (y), mean (SD)	Sex (male), *N* (%)	BMI (kg/m^2^), mean (SD)	DM, *N* (%)	ASCVD, *N* (%)	Triglycerides, mean (SD) mg/L	LDL‐C, mean (SD) mg/dL	Non‐HDL‐C, mean (SD) mg/dL	HBA1c %, Mean (SD)	VLDL, mean (SD) mg/dL	Apolipoprotein C‐III, mean (SD) mg/dL	Apolipoprotein B, mean (SD) mg/dL	Medications, *N* (%)				
															Statins	PCSK9 inhibitor	Ezetimibe	Fibrates	Omega‐3 fatty acid
Bergmark et al. 2025 (Essence–TIMI 73b)	Olezarsen 50 mg	254	63 (8.95)	157 (61.8)	31.97 (5.5)	151 (59.4)	115 (45.3)	243.77 (91.71)	83 (40.26)	126.27 (45.11)	6.37 (1.04)	41.4 (16.63)	15.43 (4.85)	90.97 (27.74)	200 (78.7)	11 (4.3)	43 (16.9)	55 (21.7)	65 (25.6)
	Olezarsen 80 mg	766	63.33 (10.4)	460 (60.05)	31.6 (4.92)	468 (61.1)	307 (40.1)	244.93 (85.04)	82.77 (35.13)	127 (41.59)	6.3 (0.97)	42.57 (15.08)	15.5 (4.01)	91.23 (25.99)	622 (81.2)	38 (5)	127 (16.6)	172 (22.5)	172 (22.5)
	Placebo	329	63 (10.42)	189 (57.44)	31.7 (5.29)	190 (57.8)	128 (38.9)	247.33 (85.63)	90.17 (38.35)	134.5 (45.42)	6.3 (1.12)	42.5 (17.5)	15.63 (4.54)	96.4 (28.52)	265 (80.5)	13 (4)	59 (17.9)	82 (24.9)	64 (19.5)
Bergmark et al. 2024 (Bridge–TIMI 73a)	Olezarsen 50 mg	58	62.67 (12.92)	34 (58.62)	33.67 (6.61)	37 (64)	16 (28)	248 (113.27)	83.1 (35.35)	156.5 (40.29)	6.5 (1.06)	45.83 (21.67)	15.53 (5.5)	90.43 (22.43)	49 (84)	3 (5)	5 (9)	5 (9)	12 (21)
	Olezarsen 80 mg	57	61 (11.41)	40 (70.17)	32.2 (6.85)	38 (67)	12 (21)	259.5 (135.38)	81.5 (30.42)	128.67 (30.42)	6.4 (0.91)	43.57 (19.55)	14.27 (4.87)	92.67 (22.06)	46 (81)	1 (2)	2 (4)	9 (16)	14 (25)
	Placebo	39	64 (10.01)	15 (38.46)	33.87 (5.54)	30 (77)	6 (15)	251.1 (50.03)	82.67 (32.71)	135.33 (39.64)	6.63 (1)	46.17 (19.24)	15.9 (4.23)	95.5 (28.09)	32 (82)	1 (3)	3 (8)	11 (28)	8 (21)
Stroes et al. 2024 (Balance)	Olezarsen 50 mg	21	43.2 (12.1)	6 (29)	22.4 (3.5)	3 (14)	NA	2684 (1235)	17.6 (8.5)	307.6 (101.8)	NA	NA	27.7 (10.5)	65.2 (13.5)	4 (19)	NA	NA	8 (38)	6 (29)
	Olezarsen 80 mg	22	47.7 (13.3)	11 (50)	25.1 (6)	7 (32)	NA	2613 (1499)	22.8 (14.1)	262.9 (100.4)	NA	NA	27.5 (11.6)	58.4 (17.2)	5 (23)	NA	NA	11 (50)	12 (55)
	Placebo	23	44 (14.7)	11 (48)	24.2 (4.1)	6 (26)	NA	2596 (1256)	16.7 (8.4)	271.3 (113.3)	NA	NA	27.7 (11.7)	59.7 (18.9)	7 (30)	NA	NA	11 (48)	7 (30)
Tardif et al. 2022 (Olezarsen Study)	Olezarsen 50 mg	22	62.9 (7.40)	15 (68.2)	32.8 (4.14)	12 (54.5)	19 (86.4)	268.4 (85.1)	76.8 (20.8)	130.1 (31)	6.42 (0.9)	56.6 (26.7)	15.7 (3.3)	88.5 (14.3)	19 (86.4)	2 (9.1)	3 (13.6)	10 (45.5)	
	Placebo	24	64.6 (7.93)	20 (83.3)	32.1 (4.18)	17 (70.8)	20 (83.3)	293.8 (86.7)	60.2 (27.2)	110.3 (24.0)	6.75 (1.06)	53.6 (11.7)	16.6 (4.5)	77.1 (19.7)	23 (95.8)	1 (4.2)	4 (16.7)	8 (33.3)	

*Note:* Data are presented as number (*N*) and percentage (%) or mean and SD.

Abbreviations: ASCVD, atherosclerotic cardiovascular disease; BMI, body mass index; DM, diabetes Mellitus; HB, haemoglobin; HDL‐C, high density lipoprotein; LDL‐C, low density lipoprotein; PCSK9, Proprotein convertase subtilisin/kexin type 9.

### 3.3 Risk of Bias

The risk of bias in the included RCTs was assessed using the ROB‐2 tool. All studies were judged to have a low risk of bias across all five domains. This was primarily due to the robust methodological quality of the trials: all were double‐blinded and employed objective outcome measures, thereby minimizing the potential for performance and detection bias. (Figure S1).

### 3.4 Primary Outcomes

#### 3.4.1 Changes in Triglycerides Level

**FIGURE 2:**
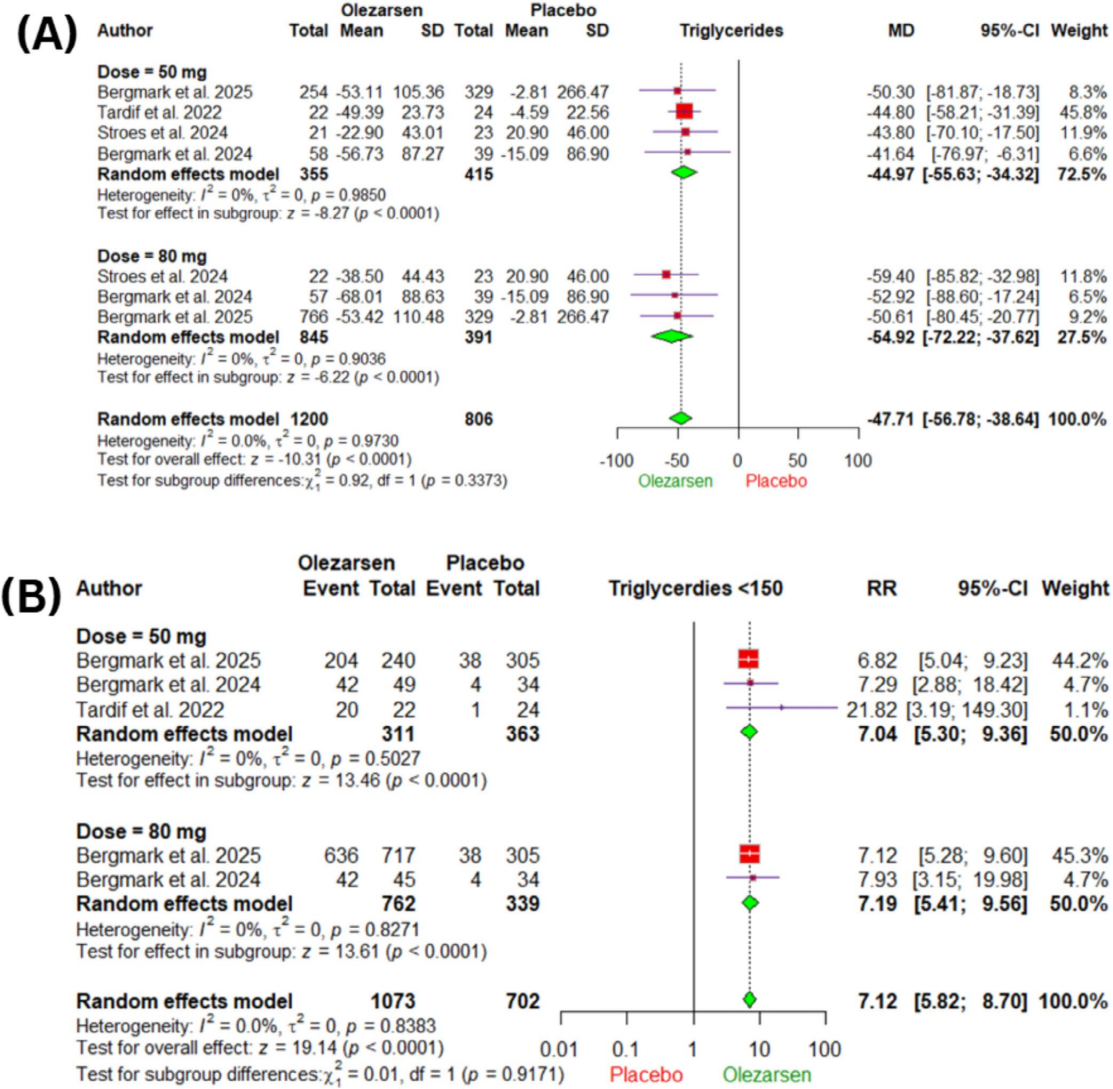
Forest plots of the primary outcomes, (A) Mean Percentage Change in Triglycerides Level, (B) Achievement of Triglycerides Level < 150 mg/dL. CI, confidence interval; MD, mean difference, RR, risk ratio.

Olezarsen was associated with a statistically significant mean percentage reduction in fasting triglycerides (MD −47.71%, 95% CI −56.78 to −38.64, *I* ^2^ = 0%, *p* < 0.0001) when compared to placebo (Figure 2a). Subgroup analysis by dose demonstrated comparable reductions with both the 50 mg (MD −44.97%, 95% CI −55.63 to −34.32, *I* ^2^ = 0%, *p* < 0.0001) and 80 mg (MD −54.92%, 95% CI −72.22 to −37.62, *I* ^2^ = 0%, *p* < 0.0001), with no statistically significant difference between the two doses (*p* = 0.33) (Figure 2a). For the 50 mg dose subgroup, reductions were observed at both follow‐up durations, with (MD −53.97%, 95% CI −69.04 to −38.90) at 6 months and (MD −44.97%, 95% CI −55.63 to −34.32) at 12 months, with no significant difference between the two follow‐up durations (*p* = 0.33) (Figure S2). In addition, for the 80 mg subgroup, reductions were observed at both follow‐up durations, with (MD −56.01%, 95% CI −71.06 to −40.95) at 6 months and (MD −54.92%, 95% CI −72.22 to −37.62) at 12 months, with no significant difference between the two follow‐up durations (*p* = 0.92) (Figure S3).

TSA showed that the cumulative Z‐curve crossed both the conventional boundary and O'Brien‐Fleming boundary, indicating evidence is conclusive for the use of olezarsen in reducing triglyceride levels. (Figure S4).

#### 3.4.2 Achievements of Triglycerides Level < 150 mg/dL

Olezarsen significantly increased the incidence of achievement of triglycerides Level < 150 mg/dL (RR 7.12, 95% CI 5.82–8.70, *I* ^2^ = 0%, *p* < 0.0001) when compared to placebo (Figure 2b). Subgroup analysis by dose revealed no significant difference (*p* = 0.91) between the 50 mg (RR 7.04, 95% CI 5.30–9.36, *I* ^2^ = 0%, *p* < 0.0001) and 80 mg (RR 7.19, 95% CI 5.41–9.56, *I* ^2^ = 0%, *p* < 0.0001) groups.

### 3.5 Secondary Outcomes

#### 3.5.1 Efficacy Outcomes

##### 3.5.1.1 Non‐HDL‐C, Apolipoprotein C‐III, and LDL‐C Levels

**FIGURE 3:**
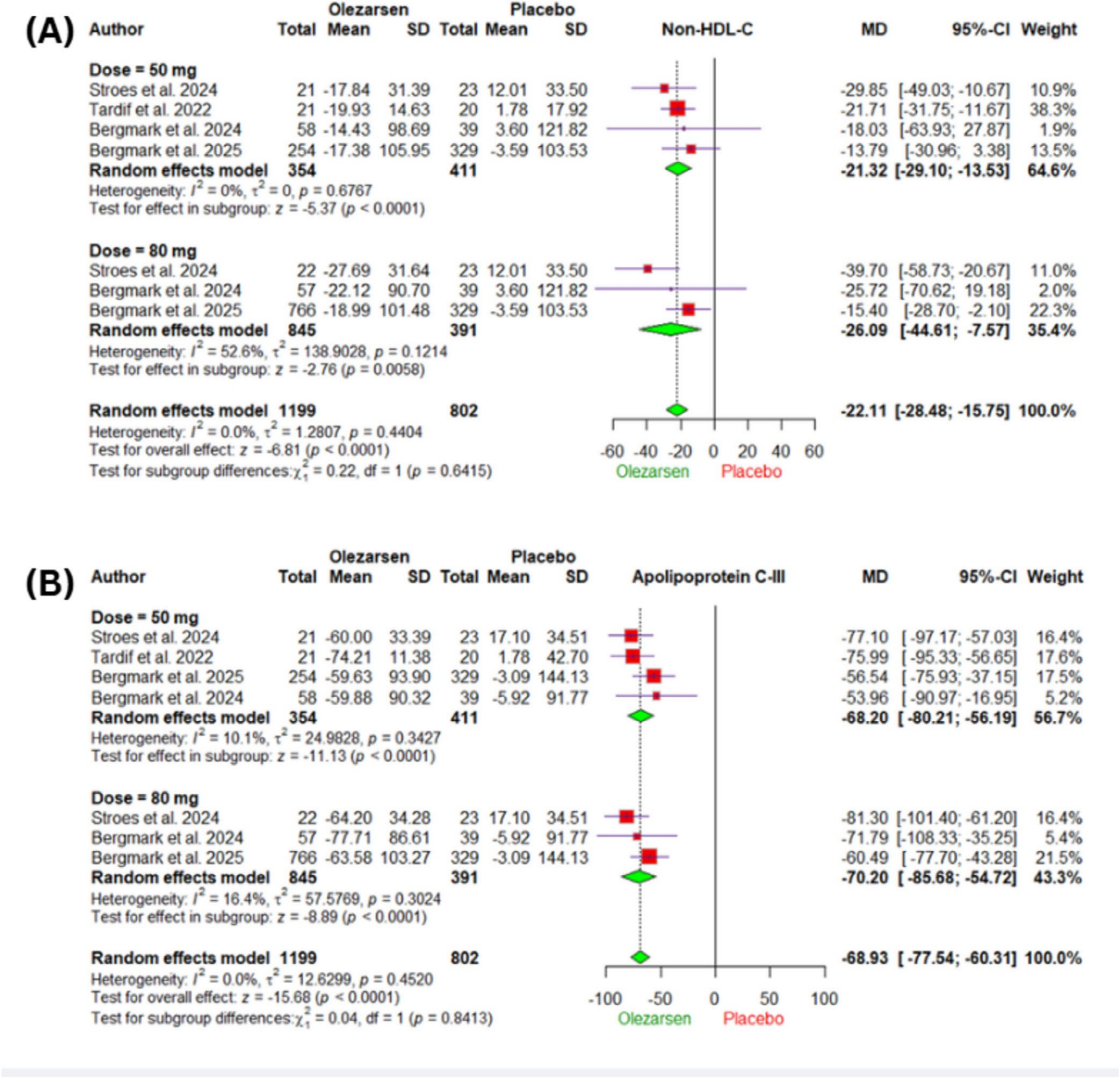
Forest plots of the secondary efficacy outcomes. (A) mean percentage change in non‐HDL‐C, (B) mean percentage change in apolipoprotein C III. CI, confidence interval; HDL‐C, high density lipoprotein cholesterol, MD, mean difference.

Olezarsen was associated with a significant reduction of mean percentage of non‐HDL‐C (MD −22.11%, 95% CI −28.48 to −15.75, *I* ^2^ = 0%, *p* < 0.0001) (Figure 3a) and ApoC‐III (MD −68.93%, 95% CI −77.54 to −60.31, *I* ^2^ = 0%, *p* < 0.0001) (Figure 3b) compared to placebo. Subgroup analysis of non‐HDL‐C by dose showed no significant difference (*p* = 0.64) between the 50 mg (MD −21.32%, 95% CI −29.10 to −13.53, *I* ^2^ = 0%, *p* < 0.0001) and 80 mg (MD −26.09%, 95% CI −44.61 to −7.57, *I* ^2^ = 52.6%, *p* = 0.005) groups (Figure 3a). Within the 50 mg subgroup, reductions were consistent at both follow‐up durations, with (MD −20.56%, 95% CI −27.87 to −13.25) at 6 months and (MD −20.83%, 95% CI −34.23 to −7.43) at 12 months, with no significant difference between them (*p* = 0.97) (Figure S5). Similarly, for the 80 mg subgroup, no significant difference (*p* = 0.71) was observed between the 6 months (MD −22.23%, 95% CI −30.88 to −13.58) and 12 months (MD −26.09%, 95% CI −44.61 to −7.57) follow‐ups (Figure S6). Subgroup analysis by olezarsen dose revealed consistent reductions in ApoC‐III levels with both the 50 mg (MD −68.20%, 95% CI −80.21 to −56.19; *I* ^2^ = 10.1%, *p* < 0.0001) and 80 mg (MD −70.20%, 95% CI −85.68 to −54.72; *I* ^2^ = 16.4%, *p* < 0.0001) doses, with no significant subgroup difference (*p* = 0.84) (Figure 3b). In the 50 mg subgroup, reductions in ApoC‐III levels were comparable at 6 and 12 months, with MD −66.66% (95% CI −76.53 to −56.79) at 6 months and MD −64.57% (95% CI −80.53 to −48.60) at 12 months, with no significant difference between time points (*p* = 0.82) (Figure S7). Similarly, in the 80 mg subgroup, no significant difference was observed between the 6‐ and 12‐month follow‐ups (*p* = 0.88), with (MD −68.85%, 95% CI −78.22 to −59.48) at 6 months and (MD −70.20%, 95% CI −85.68 to −54.72) at 12 months (Figure S8).

On the other hand, there was no statistically significant difference between olezarsen and placebo in the reduction of mean percentage of LDL‐C (MD 3.30%, 95% CI −4.91 to 11.52, *I* ^2^ = 0%, *p* = 0.43) (Figure S9). Subgroup analysis by dose showed no significant difference between the 50 and 80 mg groups (*p* = 0.49), with MD 5.79% (95% CI −5.07 to 16.65; *I* ^2^ = 0%; *p* = 0.29) for the 50 mg dose and MD −0.03% (95% CI −12.60 to 12.54, *I* ^2^ = 0%, *p* = 0.99) for the 80 mg dose (Figure S9). In the 50 mg subgroup, reductions in LDL‐C levels were comparable at 6 and 12 months, with MD 4.78% (95% CI −6.24 to 15.81) at 6 months and MD −0.73% (95% CI −16.67 to 15.22) at 12 months, with no significant difference between time points (*p* = 0.57) (Figure S10). Similarly, in the 80 mg subgroup, reductions were similar at 6 and 12 months: 6 months: (MD −1.28%, 95% CI −14.01 to 11.44); 12 months: (MD −0.87%, 95% CI −13.52 to 11.79), the difference between follow‐ups was not statistically significant (*p* = 0.96) (Figure S11).

TSA of non‐HDL‐C and ApoC‐III showed that the cumulative Z‐curve crossed both the conventional boundary and O'Brien‐Fleming boundary, indicating evidence is conclusive for the use of olezarsen over placebo in reducing non‐HDL‐C and ApoC‐III levels (Figures S12 and S13). In contrast, the TSA for LDL‐C did not cross any boundary, indicating inconclusive evidence and the need for further studies with a sample size of 13,082 (Figure S14).

##### 3.5.1.2 Other Lipid Biomarkers

Compared to placebo, olezarsen was associated with a significant increase of mean percentage of HDL‐C (MD 35.13%, 95% CI 27.30 to 42.96, *I* ^2^ = 0%, *p* < 0.0001) (Figure S15). Subgroup analysis showed no statistically significant difference between doses (*p* = 0.30), with MD 32.55% (95% CI 24.75 to 40.36, *I* ^2^ = 0%, *p* < 0.0001) for the 50 mg dose and MD 41.33% (95% CI 26.46 to 56.20, *I* ^2^ = 0%, *p* < 0.0001) for the 80 mg dose (Figure S15). Within the 50 mg subgroup, increases were consistent at both follow‐up durations (MD 67.03%, 95% CI 4.85 to 129.21) at 6 months vs. (MD 41.46%, 95% CI 23.05 to 59.86) at 12 months, with no significant subgroup difference (*p* = 0.43) (Figure S16). For the 80 mg subgroup, increases were observed at both follow‐up durations, with MD 90.00% (95% CI 0.72 to 179.27) at 6 months and MD 41.33% (95% CI 26.46 to 56.20) at 12 months, with no significant difference between the two follow‐up durations (*p* = 0.29) (Figure S17).

TSA of HDL‐C showed that the cumulative Z‐curve crossed both the conventional boundary and O'Brien‐Fleming boundary, indicating evidence is conclusive (true positive) (Figure S18).

In addition, olezarsen was associated with a significant reduction of mean percentage of VLDL‐C (MD −48.52%, 95% CI −57.16 to −39.87, *I* ^2^ = 0%, *p* < 0.0001) (Figure S19), remnant cholesterol (MD −48.53%, 95% CI −57.89 to −39.16, *I* ^2^ = 0%, *p* < 0.0001) (Figure S20), and ApoB (MD −10.67%, 95% CI −16.83 to −4.51, *I* ^2^ = 0%, *p* = 0.0007) (Figure S21). Subgroup analysis for VLDL‐C, remnant cholesterol, and ApoB showed no statistically significant differences between doses (*p* = 0.65, *p* = 0.94, and *p* = 0.97, respectively) (Figures S19–S21). For the 50 mg subgroup, subgroup analysis for VLDL‐C, remnant cholesterol, and ApoB showed no statistically significant differences between the two follow‐ups (6 and 12 months) (*p* = 0.62, *p* = 0.22, and *p* = 0.83, respectively) (Figures S22–S24). Similarly, for the 80 mg subgroup, no significant differences were observed between the two follow‐up durations for these outcomes (*p* > 0.05 for all comparisons) (Figures S25–S27).

#### 3.5.2 Safety Outcomes

##### 3.5.2.1 Adverse Events

There was no statistically significant difference between olezarsen and placebo regarding the incidence of any AEs (RR 1.01, 95% CI 0.96–1.07, *I* ^2^ = 13.9%, *p* = 0.67) (Figure S28), AEs leading to drug discontinuation (RR 1.30, 95% CI 0.87–1.94, *I* ^2^ = 0%, *p* = 0.19) (Figure S29), serious AEs (RR 0.92, 95% CI 0.62–1.37, *I* ^2^ = 30.2%, *p* = 0.67) (Figure S30), serious AEs leading to drug discontinuation (RR 1.28, 95% CI 0.58–2.81, *I* ^2^ = 0%, *p* = 0.54) (Figure S31), and possible hypersensitivity reaction (RR 0.91, 95% CI 0.60–1.37, *I* ^2^ = 0%, *p* = 0.64) (Figure S32). Subgroup analysis for any AEs, AEs leading to drug discontinuation, serious AEs, serious AEs leading to drug discontinuation, and possible hypersensitivity reaction showed no statistically significant differences between doses (*p* = 0.86, *p* = 0.75, *p* = 0.73, *p* = 0.34, and *p* = 0.50, respectively).

On the other hand, olezarsen was associated with a significant increase in the incidence of injection site reaction (RR 5.29, 95% CI 2.62–10.71, *I* ^2^ = 33.8%, *p* < 0.0001) when compared to placebo. Subgroup analysis revealed no statistically significant differences between doses (*p* = 0.88) (Figure S33).

##### 3.5.2.2 Acute Pancreatitis

Episodes of acute pancreatitis were evaluated in three trials. Overall, olezarsen significantly reduced the risk of acute pancreatitis compared with placebo (RR 0.26, 95% CI 0.07–0.91, *I* ^2^ = 0%, *p* = 0.035), while reductions observed with the 50 mg (RR 0.21, 95% CI 0.03–1.28, *I* ^2^ = 0%, *p* = 0.08) and 80 mg doses (RR 0.37, 95% CI 0.05–2.83, *I* ^2^ = 13.7%, *p* = 0.33) did not reach statistical significance, with no significant differences between dose subgroups (*p* = 0.67) (Figure S34).

##### 3.5.2.3 Abnormal Laboratory Findings

There was no statistically significant difference between olezarsen and placebo regarding the incidence of decrease in eGFR ≥ 30% (RR 0.73, 95% CI 0.49–1.07, *I* ^2^ = 0%, *p* = 0.10) (Figure S35), urinary protein: creatinine ratio ≥ 1000 (RR 0.79, 95% CI 0.44–1.40, *I* ^2^ = 0%, *p* = 0.41) (Figure S36), platelet count < 100,000/μl (RR 2.24, 95% CI 0.99–5.05, *I* ^2^ = 0%, *p* = 0.052) (Figure S37), ALT or AST level ≥ 5× ULN (RR 1.38, 95% CI 0.33–5.73, *I* ^2^ = 0%, *p* = 0.65) (Figure S38), and total bilirubin level ≥ 2× ULN (RR 0.79, 95% CI 0.15–4.13, *I* ^2^ = 0%, *p* = 0.78) (Figure S39). Subgroup analysis for decrease in eGFR ≥ 30%, urinary protein: creatinine ratio ≥ 1000, platelet count < 100,000/μL, ALT or AST level ≥ 5× ULN, and total bilirubin level ≥ 2× ULN showed no statistically significant differences between doses (*p* = 0.94, *p* = 0.79, *p* = 0.54, *p* = 0.56, and *p* = 0.63 respectively).

Overall, olezarsen was associated with a significantly higher risk of ALT or AST elevations ≥ 3× ULN compared with placebo (RR 2.33, 95% CI 1.01–5.36, *I* ^2^ = 0%, *p* = 0.046), while the increases observed with 50 mg (RR 2.64, 95% CI 0.78–8.88, *p* = 0.11) and 80 mg (RR 2.09, 95% CI 0.66–6.55, *p* = 0.20) doses were not statistically significant due to wide confidence intervals, with no significant differences between dose subgroups (*p* = 0.78) (Figure S40).

In addition, olezarsen was associated with a significant increase in HbA1c (MD 0.21, 95% CI 0.09–0.33, I^2^ = 13.6%, *p* = 0.0009) when compared to placebo (Figure S41). Subgroup analysis showed no significance between doses (*p* = 0.92). Also, subgroup analysis according to follow‐up (6 and 12 months) showed non‐significant differences for both 50 and 80 mg subgroups (all *p* > 0.05) (Figures S42 and S43).

### 3.6 Certainty of Evidence

The overall findings of the GRADE assessment are summarized in Table S3. The GRADE assessment indicated high‐certainty evidence supporting the effects of olezarsen in reducing triglycerides, non‐HDL‐C, VLDL‐C, ApoB, and ApoC‐III, along with an increase in HDL‐C levels. The evidence for LDL‐C was rated as moderate certainty due to imprecision. Regarding safety outcomes, the certainty of evidence was high for any AEs and moderate for serious AEs, acute pancreatitis, and liver enzyme elevations (ALT or AST ≥ 3× ULN), mainly due to imprecision. Overall, the certainty of evidence ranged from high for lipid efficacy outcomes to moderate for safety outcomes, mainly downgraded due to imprecision and limited event numbers.

## 4 Discussion

In this meta‐analysis, we tested the hypothesis that olezarsen favourably modifies atherogenic lipids and related outcomes compared with placebo. Across four randomized trials, fasting triglycerides fell by about half, and patients were about seven times more likely to reach triglycerides below 150 mg/dL. Non‐HDL cholesterol decreased by roughly one‐fifth, ApoC‐III dropped by about two‐thirds, and both VLDL‐C and remnant cholesterol were roughly half as high as control. ApoB declined by about one‐tenth, HDL‐C rose by about one‐third, and LDL‐C showed no clear difference. Safety was broadly comparable for overall and serious AEs, though injection‐site reactions were about fivefold higher, liver enzymes meeting the three‐times‐upper‐limit criterion occurred at roughly double the odds, and HbA1c edged up by about two‐tenths of a percentage point. Effects appeared consistent across the lower and higher dose regimens and across half‐year and 1‐year follow‐up, and sequential monitoring indicated that the evidence base for key lipid endpoints is already sufficiently informative.

Olezarsen is a hepatocyte‐targeted antisense oligonucleotide that lowers triglycerides by suppressing hepatic ApoC‐III expression. It belongs to the same therapeutic class as plozasiran, which achieves ApoC‐III reduction through a liver‐targeted small interfering RNA (siRNA) mechanism. Both agents have demonstrated substantial reductions in triglycerides and ApoC‐III levels in clinical studies involving severe hypertriglyceridemia, including FCS, as well as broader hypertriglyceridemia populations. The main distinction between these therapies lies in their molecular platforms (antisense oligonucleotide versus siRNA), which may influence dosing schedules and tolerability profiles [20, 21, 23]. In contrast, therapies targeting ANGPTL3, such as the siRNA agent zodasiran, lower triglyceride‐rich lipoproteins by inhibiting hepatic ANGPTL3 and may also reduce other atherogenic lipoproteins in mixed dyslipidemia. This mechanism is conceptually similar to the approved ANGPTL3 monoclonal antibody evinacumab, which lowers LDL‐C and triglycerides through ANGPTL3 inhibition, although it requires intravenous administration compared with the typically subcutaneous delivery used for siRNA‐based therapies [24].

The most notable finding is how strongly ApoC‐III inhibition moves patients out of the high‐triglyceride range. In our study, olezarsen reduced fasting triglycerides by roughly half and made achieving levels below 150 mg/dL almost seven times more likely. This aligns with many studies, including meta‐analyses and major randomized trials. The BRIDGE–TIMI 73a study in patients with high‐risk hypertriglyceridemia showed large and sustained percentage reductions across doses for up to 12 months, while a phase 3 trial in FCS demonstrated similarly profound triglyceride lowering compared with placebo. These data confirm the benefit of olezarsen in both common polygenic and rare monogenic hypertriglyceridemia [8, 20]. Serag et al. in their meta‐analysis also reported similar findings, with significant reductions in triglyceride levels (MD: −76.11 mg/dL) [4]. Comparable results were also reported in the meta‐analysis of all ApoC‐III inhibitors by de Souza and colleagues, who demonstrated a significant reduction in triglycerides 60% (SMD: −60.56%) [25]. From the pathophysiological standpoint, ApoC‐III normally slows the breakdown and hepatic clearance of triglyceride‐rich lipoproteins (chylomicrons and VLDL‐C) by inhibiting lipoprotein lipase and blocking remnant uptake. Inhibiting ApoC‐III removes this brake, enhancing lipolysis and hepatic uptake, which rapidly reduces circulating triglyceride concentrations [26].

Beyond triglycerides, our analysis showed a consistent improvement across the triglyceride‐rich lipoprotein (TRL) pathway. Levels of non‐HDL‐C decreased, VLDL‐C and remnant cholesterol were reduced by about half, and ApoC‐III dropped markedly. HDL‐C increased notably, while LDL‐C remained largely unchanged. These findings align with recent randomized data; in the BRIDGE–TIMI 73a trial, olezarsen lowered non‐HDL‐C and ApoB, and in plozasiran trials, non‐HDL‐C decreased with little change in ApoB, suggesting that triglyceride content within lipoproteins is reduced more than particle number [8, 27]. Moreover, de Souza et al. demonstrated a significant increase in HDL‐C levels, with an improvement of approximately 44% (SMD: 43.92%), and a notable reduction in non‐HDL‐C levels of roughly 27% (SMD: −27.49%) [25]. Additionally, Serag and colleagues reported a comparable pattern in their study, demonstrating an increase in HDL‐C levels (MD = +22.60 mg/dL) and a decrease in VLDL‐C levels (MD = −54.95 mg/dL) [4]. This pattern can be explained by the role of ApoC‐III. When its inhibitory effect is removed, lipolysis of large TRLs is enhanced, leading to faster breakdown of VLDL‐C and remnants. As these cholesterol‐rich particles diminish, non‐HDL‐C falls accordingly. Meanwhile, HDL‐C may rise due to the transfer of surface components from shrinking TRLs, while LDL‐C remains stable because hepatic production of ApoB‐containing particles and their conversion to LDL are less affected than triglyceride removal [26, 28].

A meaningful clinical outcome of ApoC‐III inhibition is the reduction in pancreatitis events. In our analysis, patients receiving olezarsen experienced fewer episodes of acute pancreatitis compared with placebo. Similarly, the phase 3 trial in FCS showed a significant drop in pancreatitis incidence among those treated with olezarsen, and Nordestgaard et al. in their recent reviews now stress the role of ApoC‐III inhibitors as promising options to reduce pancreatitis risk in severe hypertriglyceridemia [20, 29]. Notably, consistent findings have also been reported with plozasiran in the PALISADE trial, where targeting ApoC‐III via an siRNA platform was associated with a reduced risk of acute pancreatitis, supporting a class‐wide effect of ApoC‐III inhibition [30]. The mechanism is clear: when plasma chylomicrons are excessively elevated, pancreatic capillaries become exposed to high concentrations of free fatty acids, leading to local inflammation and tissue injury. By rapidly reducing chylomicronemia, ApoC‐III blockade minimizes this lipotoxic stress and lowers the likelihood of an acute pancreatitis attack [28, 30].

One of the most reassuring aspects of ApoC‐III inhibition is its safety. Our pooled analysis showed that olezarsen did not increase overall or serious AEs compared with placebo, although mild injection‐site reactions were more common. This finding is consistent with modern randomized studies in both hypertriglyceridemia and FCS, where tolerability was similar to placebo except for local reactions at injection sites [8, 20]. This safety pattern reflects how the drug is designed. Ligand‐conjugated antisense delivery directs activity to hepatocytes, the main site of ApoC‐III production, so the effect stays concentrated in the liver. Subcutaneous dosing can cause mild local irritation but rarely leads to systemic side effects. By contrast, the older agent volanesorsen, though effective in lowering lipids, was limited by dose‐related thrombocytopenia, showing how advances in drug chemistry and targeted delivery have improved the safety of ApoC‐III inhibitors [31, 32].

A subtle yet important aspect of ApoC‐III inhibition involves its laboratory profile. In our meta‐analysis, olezarsen was associated with small biochemical changes, mildly higher rates of ALT and AST elevations (≥ 3× ULN) and a slight increase in HbA1c, while renal function and significant proteinuria were comparable to placebo. Platelet counts showed a nonsignificant downward trend without any clear safety signal. These findings are consistent with Bergmark et al. trial, who also reported minor enzyme elevations, stable renal parameters, and no clinically meaningful worsening of glycemic control, though continued monitoring remains advisable [8]. These modest biochemical findings are consistent with how hepatocyte‐targeted therapies influence lipid metabolism. Because RNA‐targeted therapies act directly in hepatocytes, they can transiently affect hepatic protein synthesis and lipid processing, explaining temporary rises in liver enzymes as the liver adapts to altered triglyceride metabolism. The small increase in HbA1c seen in pooled analyses may reflect changes in fatty acid availability and hepatic energy substrate preference; however, randomized data to date show no consistent evidence of clinically relevant deterioration in glucose control [28].

### 4.1 Clinical Implications

Our findings suggest that olezarsen can serve as a useful add‐on therapy for adults with persistent hypertriglyceridemia who remain above target levels despite diet, secondary‐cause correction, and standard lipid‐lowering treatment. The marked triglyceride reduction and higher chance of achieving levels below 150 mg/dL point to its potential for lowering pancreatitis risk and improving overall lipid control.

In clinical practice, olezarsen may be most appropriate for patients with sustained high triglycerides, recurrent or impending pancreatitis, and elevated non‐HDL‐C or remnant cholesterol. The observed decreases in non‐HDL‐C, VLDL‐C, remnant cholesterol, and ApoB further support its role in comprehensive cardiovascular risk reduction. Physicians can aim for clear targets (such as triglycerides < 150 mg/dL and improved non‐HDL‐C) while maintaining lifestyle measures and background therapy.

Routine safety monitoring remains important. Although adverse and serious events were similar to placebo, clinicians should monitor for injection‐site reactions and periodic lab changes, particularly liver enzymes and glycemic indices, about 8–12 weeks after initiation and at regular follow‐up. Reinforcing proper injection technique and checking platelet and renal function as part of standard lipid‐clinic protocols are reasonable precautions.

Both 50 mg and 80 mg doses produced comparable lipid improvements over 6–12 months, allowing flexible dosing based on patient preference and tolerance. Tracking early triglyceride response (e.g., achieving < 150 mg/dL) can guide continued therapy and individualized decisions.

Because current evidence does not yet address major cardiovascular or long‐term outcomes, olezarsen should be used within a broader preventive framework, including control of blood pressure, glycemia, smoking, and LDL‐related risk. Ongoing trials will clarify its impact on clinical endpoints and help refine patient selection and monitoring strategies.

### 4.2 Strengths and Limitations

Our study has several methodological strengths that enhance the reliability and clinical relevance of its findings. This meta‐analysis included only RCTs, all of which were double‐blinded and multicentre, ensuring high methodological quality and minimizing bias. The trials were conducted across multiple regions, including North America and Europe, and involved patients with hypertriglyceridemia at high cardiovascular risk, enhancing external validity. We conducted subgroup analyses according to olezarsen dose (50 mg vs. 80 mg) and follow‐up duration (6 vs. 12 months) to examine dose–response and time‐dependent effects. Furthermore, TSA was applied to assess conclusiveness, and the GRADE approach was used to rate the certainty of the evidence, strengthening the robustness and interpretability of the results.

Despite these strengths, the number of included trials was small, and the total sample size remained modest, limiting the statistical power for rare outcomes. The follow‐up period across trials ranged only from 6 to 12 months, preventing assessment of long‐term cardiovascular benefits or safety outcomes. In addition, the included trials were not designed or powered to assess major adverse cardiovascular events (MACE), and no study reported MACE stratified by achieved LDL‐C or ApoB levels; therefore, we were unable to generate plots examining the relationship between changes in LDL‐C or ApoB and cardiovascular outcomes, and any inference regarding their impact on MACE remains speculative. Although subgroup analyses were conducted, differences in trial design, dosing schedules, and patient characteristics may have introduced unmeasured variability. Data on clinical endpoints such as major cardiovascular events and mortality were limited, and some laboratory abnormalities (e.g., elevated transaminases and HbA1c) warrant longer‐term safety monitoring. Publication bias cannot be fully excluded, and additional large‐scale outcome‐driven trials are required to validate these findings and determine their clinical significance.

## 5 Conclusion

In this meta‐analysis of randomized trials, olezarsen produced strong and consistent improvements in atherogenic lipids, cutting triglycerides by about half and lowering non‐HDL‐C, VLDL‐C, remnant cholesterol, and ApoC‐III without increasing serious AEs. Mild enzyme and glucose shifts were small and clinically manageable. These findings support olezarsen as a promising add‐on option for patients with persistent hypertriglyceridemia despite standard therapy, helping more patients reach safer triglyceride levels that may reduce pancreatitis and overall lipid‐related risk. Longer outcome trials are still needed to determine its long‐term cardiovascular benefits.

## Author Contributions

**A.E.:** conceptualization, project administration, software, methodology, visualization, investigation, data curation and writing – original draft preparation. **A.A.:** methodology, investigation and writing – original draft preparation. **H.A.:** data curation, formal analysis, and writing – original draft preparation. **A.F.G. A.M.A., and A.A.A.:** investigation, data collection, and resources. **H.A.A.‐H., M.J.A., and. S.A.A.:** writing – original draft preparation. **A.S.:** supervision and writing – review and editing. All authors have read and agreed to the final version of the manuscript.

## Funding

The authors have nothing to report.

## Disclosure

The authors confirm that no paper mill or artificial intelligence was used.

## Ethics Statement

The authors have nothing to report.

## Consent

The authors have nothing to report.

## Conflicts of Interest

The authors declare no conflicts of interest.

## Supporting information


**Table S1:** PRISMA 2020 checklist.
**Table S2:** Search strategy and literature search.
**Table S3:** Grading of recommendations assessment, development, and evaluation.
**Figure S1:** Overview of the risk of bias of the included randomized controlled trials.
**Figure S2:** Forest plot of subgroup analysis by follow‐up for changes in triglycerides level for 50 mg dose.
**Figure S3:** Forest plot of subgroup analysis by follow‐up for changes in triglycerides level for 80 mg dose.
**Figure S4:** Trial sequential analysis (TSA) of triglycerides change.
**Figure S5:** Forest plot of subgroup analysis by follow‐up for changes in Non‐HDL‐C level for 50 mg dose.
**Figure S6:** Forest plot of subgroup analysis by follow‐up for changes in Non‐HDL‐C level for 80 mg dose.
**Figure S7:** Forest plot of subgroup analysis by follow‐up for changes ApoC‐III level for 50 mg dose.
**Figure S8:** Forest plot of subgroup analysis by follow‐up for changes in ApoC‐III level for 80 mg dose.
**Figure S9:** Forest plot of subgroup analysis by dose for changes in LDL‐C level.
**Figure S10:** Forest plot of subgroup analysis by follow‐up for changes in LDL‐C level for 50 mg dose.
**Figure S11:** Forest plot of subgroup analysis by follow‐up for changes in LDL‐C level for 80 mg dose.
**Figure S12:** Trial sequential analysis (TSA) of Non‐HDL‐C change.
**Figure S13:** Trial sequential analysis (TSA) of ApoC‐III change.
**Figure S14:** Trial sequential analysis (TSA) of LDL‐C change.
**Figure S15:** Forest plot of subgroup analysis by dose for changes in HDL‐C level.
**Figure S16:** Forest plot of subgroup analysis by follow‐up for changes in HDL‐C level in 50 mg dose.
**Figure S17:** Forest plot of subgroup analysis by follow‐up for changes in HDL‐C level in 80 mg dose.
**Figure S18:** Trial sequential analysis (TSA) of HDL‐C change.
**Figure S19:** Forest plot of subgroup analysis by dose for changes in VLDL‐C.
**Figure S20:** Forest plot of subgroup analysis by dose for changes in remnant cholesterol.
**Figure S21:** Forest plot of subgroup analysis by dose for changes in ApoB.
**Figure S22:** Forest plot of subgroup analysis by follow‐up for changes of VLDL‐C in 50 mg dose.
**Figure S23:** Forest plot of subgroup analysis by follow‐up for changes of remnant cholesterol in 50 mg dose.
**Figure S24:** Forest plot of subgroup analysis by follow‐up for changes in ApoB in 50 mg dose.
**Figure S25:** Forest plot of subgroup analysis by follow‐up for changes in VLDL‐C in 80 mg dose.
**Figure S26:** Forest plot of subgroup analysis by follow‐up for changes in remnant cholesterol in 80 mg dose.
**Figure S27:** Forest plot of subgroup analysis by follow‐up for changes in ApoB in 80 mg dose.
**Figure S28:** Forest plot of any adverse events.
**Figure S29:** Forest plot of adverse events leading to drug discontinuation.
**Figure S30:** Forest plot of serious adverse events.
**Figure S31:** Forest plot of serious adverse events leading to drug discontinuation.
**Figure S32:** Forest plot of possible hypersensitivity reaction.
**Figure S33:** Forest plot of injection site reaction.
**Figure S34:** Forest plot of acute pancreatitis.
**Figure S35:** Forest plot of decrease in eGFR ≥ 30%.
**Figure S36:** Forest plot of urinary protein: creatinine ratio ≥ 1000.
**Figure S37:** Forest plot of platelet count < 100,000/μl.
**Figure S38:** Forest plot of ALT or AST level ≥ 5× ULN.
**Figure S39:** Forest plot of total bilirubin level ≥ 2× ULN.
**Figure S40:** Forest plot of ALT or AST level ≥ 3× ULN.
**Figure S41:** Forest plot of change in HBA1C.
**Figure S42:** Forest plot of subgroup analysis by follow‐up for HBA1C in 50 mg dose.
**Figure S43:** Forest plot of subgroup analysis by follow‐up for HBA1C in 80 mg dose.

## Data Availability

The authors have nothing to report.
