# Supplementary material for: Olezarsen in Hypertriglyceridemia With High Cardiac Risk: A GRADE‐Assessed Meta‐Analysis of Randomized Trials With Trial Sequential Evidence
Source: Endocrinol Diabetes Metab. 2026 Apr 20;9(3):e70220. doi: 10.1002/edm2.70220 (PMC13095862; doi:10.1002/edm2.70220)
Supplement: Supplementary file 1 — Table S1: PRISMA 2020 checklist. Table S2: Search strategy and literature search. Table S3: Grading of recommendations assessment, development, and evaluation. Figure S1: Overview of the risk of bias of the included randomized controlled trials. Figure S2: Forest plot of subgroup analysis by follow‐up for changes in triglycerides level for 50 mg dose. Figure S3: Forest plot of subgroup analysis by follow‐up for changes in triglycerides level for 80 mg dose. Figure S4: Trial sequential analysis (TSA) of triglycerides change. Figure S5: Forest plot of subgroup analysis by follow‐up for changes in Non‐HDL‐C level for 50 mg dose. Figure S6: Forest plot of subgroup analysis by follow‐up for changes in Non‐HDL‐C level for 80 mg dose. Figure S7: Forest plot of subgroup analysis by follow‐up for changes ApoC‐III level for 50 mg dose. Figure S8: Forest plot of subgroup analysis by follow‐up for changes in ApoC‐III level for 80 mg dose. Figure S9: Forest plot of subgroup analysis by dose for changes in LDL‐C level. Figure S10: Forest plot of subgroup analysis by follow‐up for changes in LDL‐C level for 50 mg dose. Figure S11: Forest plot of subgroup analysis by follow‐up for changes in LDL‐C level for 80 mg dose. Figure S12: Trial sequential analysis (TSA) of Non‐HDL‐C change. Figure S13: Trial sequential analysis (TSA) of ApoC‐III change. Figure S14: Trial sequential analysis (TSA) of LDL‐C change. Figure S15: Forest plot of subgroup analysis by dose for changes in HDL‐C level. Figure S16: Forest plot of subgroup analysis by follow‐up for changes in HDL‐C level in 50 mg dose. Figure S17: Forest plot of subgroup analysis by follow‐up for changes in HDL‐C level in 80 mg dose. Figure S18: Trial sequential analysis (TSA) of HDL‐C change. Figure S19: Forest plot of subgroup analysis by dose for changes in VLDL‐C. Figure S20: Forest plot of subgroup analysis by dose for changes in remnant cholesterol. Figure S21: Forest plot of subgroup analysis by dose for changes in ApoB. Figure [file EDM2-9-e70220-s001.docx]

**Supplemental Materials**

**Olezarsen in Hypertriglyceridemia with High Cardiac Risk: A GRADE- Assessed Meta-analysis of Randomized Trials with Trial Sequential Evidence.**

The authors have provided this supplementary material to demonstrate additional information about this study.

**Supplementary Table 1** PRISMA 2020 checklist.

| **Section and Topic** | **Item #** | **Checklist item** | **Location where item is reported** |
| --- | --- | --- | --- |
| **TITLE** | | | |
| Title | 1 | Identify the report as a systematic review. | Title page |
| **ABSTRACT** | | | |
| Abstract | 2 | See the PRISMA 2020 for Abstracts checklist. | Page 2,3 |
| **INTRODUCTION** | | | |
| Rationale | 3 | Describe the rationale for the review in the context of existing knowledge. | Page 4,5 |
| Objectives | 4 | Provide an explicit statement of the objective(s) or question(s) the review addresses. | Page 4,5 |
| **METHODS** | | | |
| Eligibility criteria | 5 | Specify the inclusion and exclusion criteria for the review and how studies were grouped for the syntheses. | Page 6,7, subsection 2.3 |
| Information sources | 6 | Specify all databases, registers, websites, organisations, reference lists and other sources searched or consulted to identify studies. Specify the date when each source was last searched or consulted. | Page 6, subsection 2.2 |
| Search strategy | 7 | Present the full search strategies for all databases, registers, and websites, including any filters and limits used. | Page 6, subsection 2.2 Sup. material, table S2 |
| Selection process | 8 | Specify the methods used to decide whether a study met the inclusion criteria of the review, including how many reviewers screened each record and each report retrieved, whether they worked independently, and if applicable, details of automation tools used in the process. | Page 7, subsection 2.4 |
| Data collection process | 9 | Specify the methods used to collect data from reports, including how many reviewers collected data from each report, whether they worked independently, any processes for obtaining or confirming data from study investigators, and if applicable, details of automation tools used in the process. | Page 7,8, subsection 2.5 |
| Data items | 10a | List and define all outcomes for which data were sought. Specify whether all results that were compatible with each outcome domain in each study were sought (e.g. for all measures, time points, analyses), and if not, the methods used to decide which results to collect. | Page 7, subsection 2.3 |
|  | 10b | List and define all other variables for which data were sought (e.g. participant and intervention characteristics, funding sources). Describe any assumptions made about any missing or unclear information. | Page 7, subsection 2.3 |
| Study risk of bias assessment | 11 | Specify the methods used to assess risk of bias in the included studies, including details of the tool(s) used, how many reviewers assessed each study and whether they worked independently, and if applicable, details of automation tools used in the process. | Page 8, subsection 2.6 |
| Effect measures | 12 | Specify for each outcome the effect measure(s) (e.g. risk ratio, mean difference) used in the synthesis or presentation of results. | Page 9,10, subsection 2.7 |
| Synthesis methods | 13a | Describe the processes used to decide which studies were eligible for each synthesis (e.g. tabulating the study intervention characteristics and comparing against the planned groups for each synthesis (item #5)). | Page 7, subsection 2.4 |
|  | 13b | Describe any methods required to prepare the data for presentation or synthesis, such as handling of missing summary statistics, or data conversions. | Page 9, subsection 2.7 |
|  | 13c | Describe any methods used to tabulate or visually display results of individual studies and syntheses. | Page 9,10, subsection 2.7 |
|  | 13d | Describe any methods used to synthesize results and provide a rationale for the choice(s). If meta-analysis was performed, describe the model(s), method(s) to identify the presence and extent of statistical heterogeneity, and software package(s) used. | Page 9,10, subsection 2.7 |
|  | 13e | Describe any methods used to explore plausible causes of heterogeneity among study results (e.g. subgroup analysis, meta-regression). | Page 10, subsection 2.7.3 |
|  | 13f | Describe any sensitivity analyses conducted to assess robustness of the synthesized results. | Page 10, subsection 2.7.3 |
| Reporting bias assessment | 14 | Describe any methods used to assess risk of bias due to missing results in a synthesis (arising from reporting biases). | Not applicable |
| Certainty assessment | 15 | Describe any methods used to assess certainty (or confidence) in the body of evidence for an outcome. | Page 8, subsection 2.6 |
| **RESULTS** | | | |
| Study selection | 16a | Describe the results of the search and selection process, from the number of records identified in the search to the number of studies included in the review, ideally using a flow diagram. | Page 11, subsection 3.1 |
|  | 16b | Cite studies that might appear to meet the inclusion criteria, but which were excluded, and explain why they were excluded. | Page 11, subsection 3.1 |
| Study characteristics | 17 | Cite each included study and present its characteristics. | Table 1 & 2, Page 11, subsection 3.2 |
| Risk of bias in studies | 18 | Present assessments of risk of bias for each included study. | Page 12, subsection 3.3 |
| Results of individual studies | 19 | For all outcomes, present, for each study: (a) summary statistics for each group (where appropriate) and (b) an effect estimates and its precision (e.g. confidence/credible interval), ideally using structured tables or plots. | Pages 12-17, subsections 3.4-3.5 |
| Results of syntheses | 20a | For each synthesis, briefly summarise the characteristics and risk of bias among contributing studies. | Page 12, subsection 3.3 |
|  | 20b | Present results of all statistical syntheses conducted. If meta-analysis was done, present for each the summary estimate and its precision (e.g. confidence/credible interval) and measures of statistical heterogeneity. If comparing groups, describe the direction of the effect. | Pages 12-17, subsections 3.4-3.5 |
|  | 20c | Present results of all investigations of plausible causes of heterogeneity among study results. | Pages 12-17, subsections 3.4-3.5 |
|  | 20d | Present results of all sensitivity analyses conducted to assess the robustness of the synthesized results. | Pages 12-17, subsections 3.4-3.5 |
| Reporting biases | 21 | Present assessments of risk of bias due to missing results (arising from reporting biases) for each synthesis assessed. | Not applicable |
| Certainty of evidence | 22 | Present assessments of certainty (or confidence) in the body of evidence for each outcome assessed. | Page 17, subsection 3.6  Table 3 |
| **DISCUSSION** | | | |
| Discussion | 23a | Provide a general interpretation of the results in the context of other evidence. | Page 18,19 |
|  | 23b | Discuss any limitations of the evidence included in the review. | Page 22, 23 |
|  | 23c | Discuss any limitations of the review processes used. | Page 22, 23 |
|  | 23d | Discuss implications of the results for practice, policy, and future research. | Page 22, 23 |
| **OTHER INFORMATION** | | | |
| Registration and protocol | 24a | Provide registration information for the review, including register name and registration number, or state that the review was not registered. | Page 6, subsection 2.1 |
|  | 24b | Indicate where the review protocol can be accessed, or state that a protocol was not prepared. | Page 6, subsection 2.1 |
|  | 24c | Describe and explain any amendments to information provided at registration or in the protocol. | Page 6, subsection 2.1 |
| Support | 25 | Describe sources of financial or non-financial support for the review, and the role of the funders or sponsors in the review. | Page 25 Section 5 |
| Competing interests | 26 | Declare any competing interests of review authors. | Page 25  Section 5 |
| Availability of data, code, and other materials | 27 | Report which of the following are publicly available and where they can be found template data collection forms; data extracted from included studies; data used for all analyses; analytic code; any other materials used in the review. | Page 25  Section 5 |

**Supplementary Table 2** Search Strategy and Literature Search.

| **Database** | **Filters** | **Access Date** | **Search Strategy** | **No of Results** |
| --- | --- | --- | --- | --- |
| **PubMed** | All Fields | 21/9/2025 | (Olezarsen OR tryngolza OR "APOC3 inhibitor" OR "apolipoprotein C-III inhibitor") AND ("Hypertriglyceridemia" OR triglyceride OR triglycerides OR hypertriglyceridemia OR hypertriglyceridaemia OR chylomicronemia OR Familial Chylomicronemia Syndrome) AND ("clinical trial" OR "controlled trial" OR randomiz* OR randomis* OR randomly OR random OR RCT OR blind*) | **87** |
| **Cochrane** | All Text | 21/9/2025 |  | **33** |
| **Scopus** | All fields | 21/9/2025 |  | **163** |
| **Web of Science** | All Fields | 21/9/2025 |  | **28** |
| **Total** | | | | **311** |

**Supplementary Table 3.** Grading of recommendations assessment, development, and evaluation.

| **Certainty assessment** | | | | | | | **Summary of findings** | | | | |
| --- | --- | --- | --- | --- | --- | --- | --- | --- | --- | --- | --- |
| **Participants (studies) Follow-up** | **Risk of bias** | **Inconsistency** | **Indirectness** | **Imprecision** | **Publication bias** | **Overall certainty of evidence** | **Study event rates (%)** | | **Relative effect (95% CI)** | **Anticipated absolute effects** | |
|  |  |  |  |  |  |  | **With Placebo** | **With Olezarsen** |  | **Risk with Placebo** | **Risk difference with Olezarsen** |
| **Triglycerides** | | | | | | | | | | | |
| 1615 (4 RCTs) | not serious | not serious | not serious | not serious | strong association | ⨁⨁⨁⨁ High | 415 | 1200 | - | 415 | MD **47.09 lower** (57.32 lower to 36.85 lower) |
| **LDL-C** | | | | | | | | | | | |
| 1606 (4 RCTs) | not serious | not serious | not serious | serious^a^ | none | ⨁⨁⨁◯ Moderate^a^ | 410 | 1196 | - | 410 | MD **4.38 higher** (5.01 lower to 13.77 higher) |
| **HDL-C** | | | | | | | | | | | |
| 1544 (3 RCTs) | not serious | not serious | not serious | not serious | dose response gradient | ⨁⨁⨁⨁ High | 388 | 1156 | - | 388 | MD **33.61 higher** (26.31 higher to 40.91 higher) |
| **Non-HDL-C** | | | | | | | | | | | |
| 1610 (4 RCTs) | not serious | not serious | not serious | not serious | none | ⨁⨁⨁⨁ High | 411 | 1199 | - | 411 | MD **22.1 lower** (29.72 lower to 14.49 lower) |
| **Apolipoprotein C-III** | | | | | | | | | | | |
| 1610 (4 RCTs) | not serious | not serious | not serious | not serious | dose response gradient | ⨁⨁⨁⨁ High | 411 | 1199 | - | 411 | MD **70.22 lower** (80.29 lower to 60.15 lower) |
| **VLDL-C** | | | | | | | | | | | |
| 1543 (3 RCTs) | not serious | not serious | not serious | not serious | none | ⨁⨁⨁⨁ High | 388 | 1155 | - | 388 | MD **49.63 lower** (59.45 lower to 39.82 lower) |
| **Apolipoprotein B** | | | | | | | | | | | |
| 1610 (4 RCTs) | not serious | not serious | not serious | not serious | none | ⨁⨁⨁⨁ High | 411 | 1199 | - | 411 | MD **10.72 lower** (17.37 lower to 4.08 lower) |
| **Any Adverse Events** | | | | | | | | | | | |
| 1743 (4 RCTs) | not serious | not serious | not serious | not serious | none | ⨁⨁⨁⨁ High | 337/455 (74.1%) | 977/1288 (75.9%) | **RR 1.01** (0.91 to 1.11) | 337/455 (74.1%) | **7 more per 1,000** (from 67 fewer to 81 more) |
| **Serious Adverse Events** | | | | | | | | | | | |
| 1743 (4 RCTs) | not serious | not serious | not serious | serious^b^ | none | ⨁⨁⨁◯ Moderate^b^ | 54/455 (11.9%) | 159/1288 (12.3%) | **RR 0.94** (0.49 to 1.80) | 54/455 (11.9%) | **7 fewer per 1,000** (from 61 fewer to 95 more) |
| **Acute Pancreatitis** | | | | | | | | | | | |
| 1569 (3 RCTs) | not serious | not serious | not serious | serious^b^ | none | ⨁⨁⨁◯ Moderate^b^ | 7/391 (1.8%) | 5/1178 (0.4%) | **RR 0.26** (0.07 to 0.91) | 7/391 (1.8%) | **13 fewer per 1,000** (from 17 fewer to 2 fewer) |
| **ALT or AST level ≥3× ULN** | | | | | | | | | | | |
| 1701 (4 RCTs) | not serious | not serious | not serious | serious^b^ | none | ⨁⨁⨁◯ Moderate^b^ | 5/449 (1.1%) | 29/1252 (2.3%) | **RR 2.33** (1.01 to 5.36) | 5/449 (1.1%) | **15 more per 1,000** (from 0 fewer to 49 more) |

**CI:** confidence interval; **MD:** mean difference; **RR:** risk ratio

#### Explanations

a. Owing to the wide 95% CI, which includes clinically important differences

b. Owing to the few numbers of events.


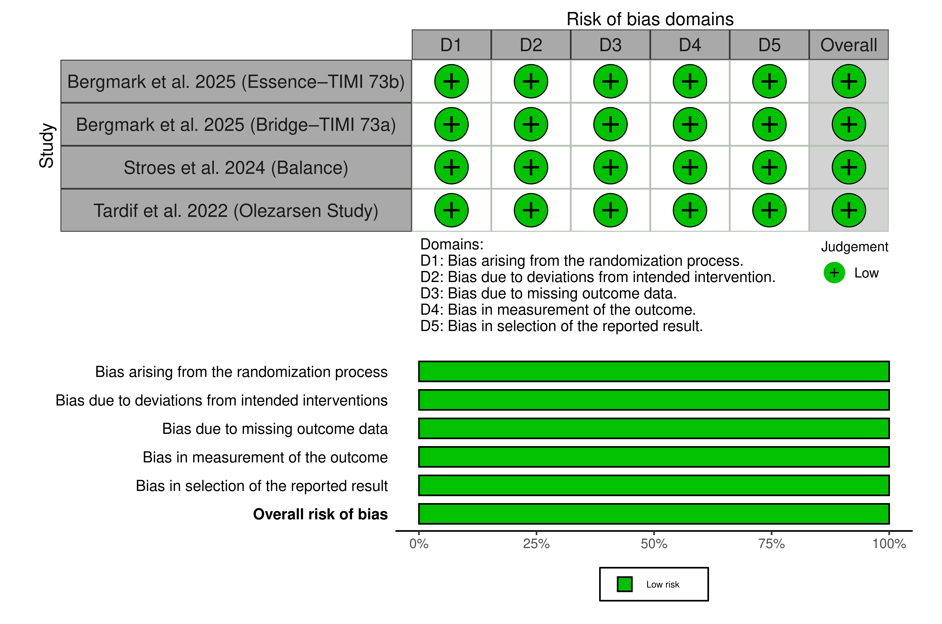


**Supplementary Fig. 1** Overview of the risk of bias of the included randomized controlled trials.

**
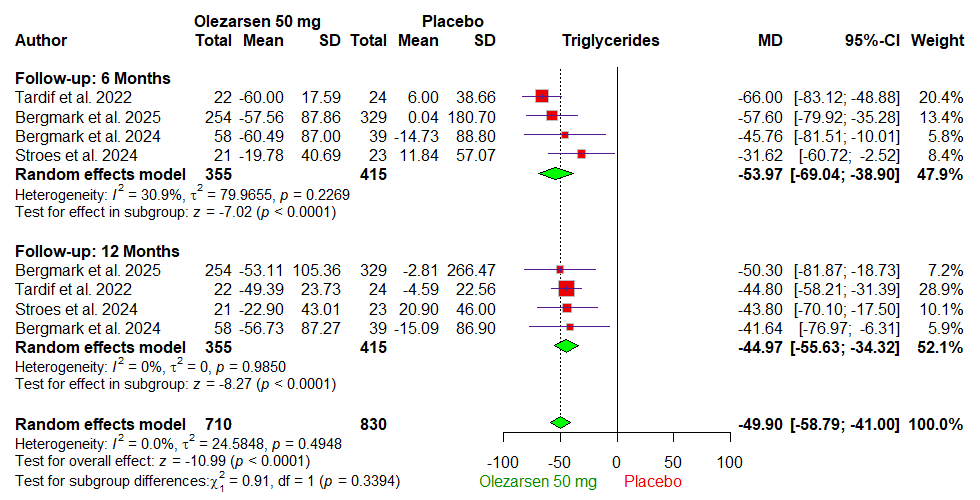
**

**Supplementary Fig. 2** Forest plot of subgroup analysis by follow-up for changes in triglycerides level for 50 mg dose.


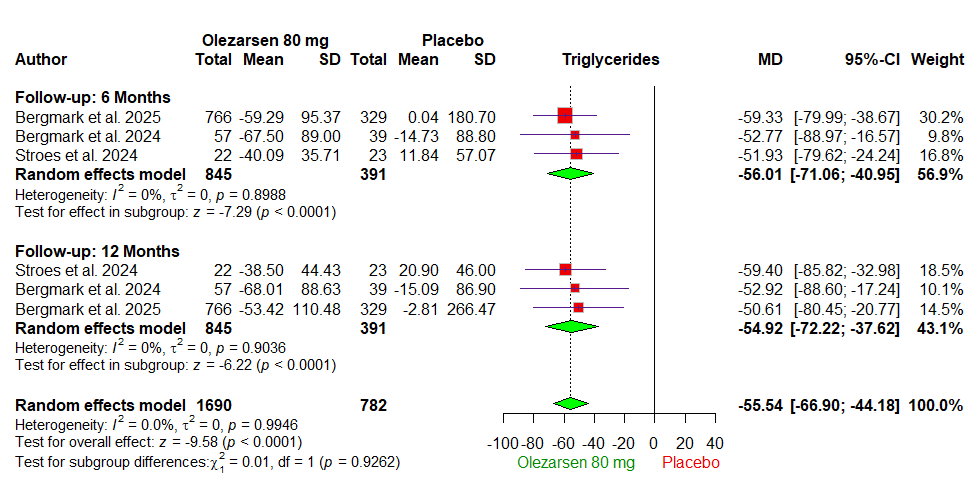


**Supplementary Fig. 3** Forest plot of subgroup analysis by follow-up for changes in triglycerides level for 80 mg dose.


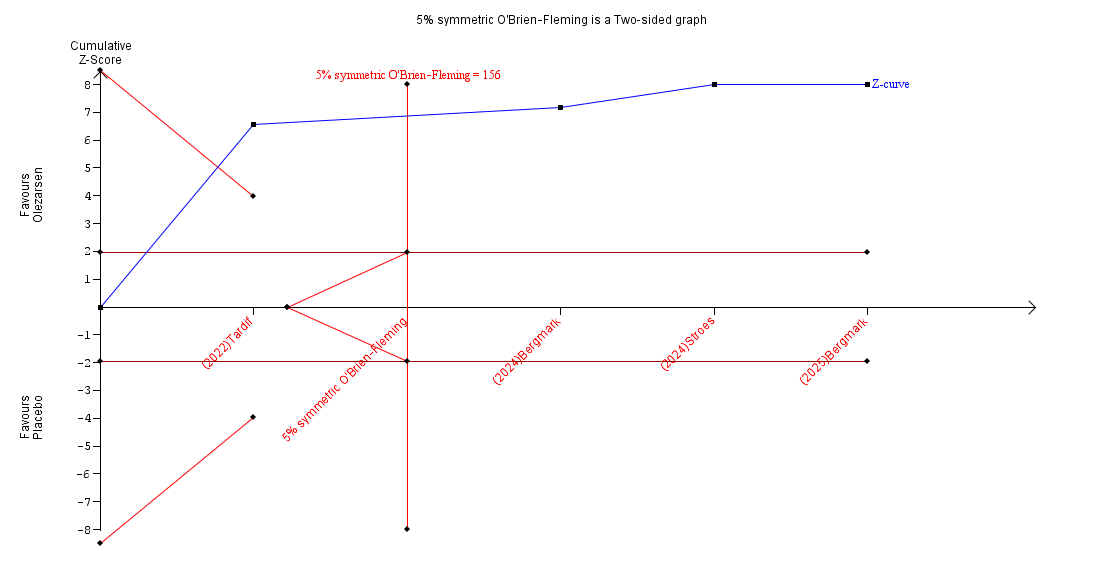


**Supplementary Fig. 4** Trial Sequential Analysis (TSA) of triglycerides change.


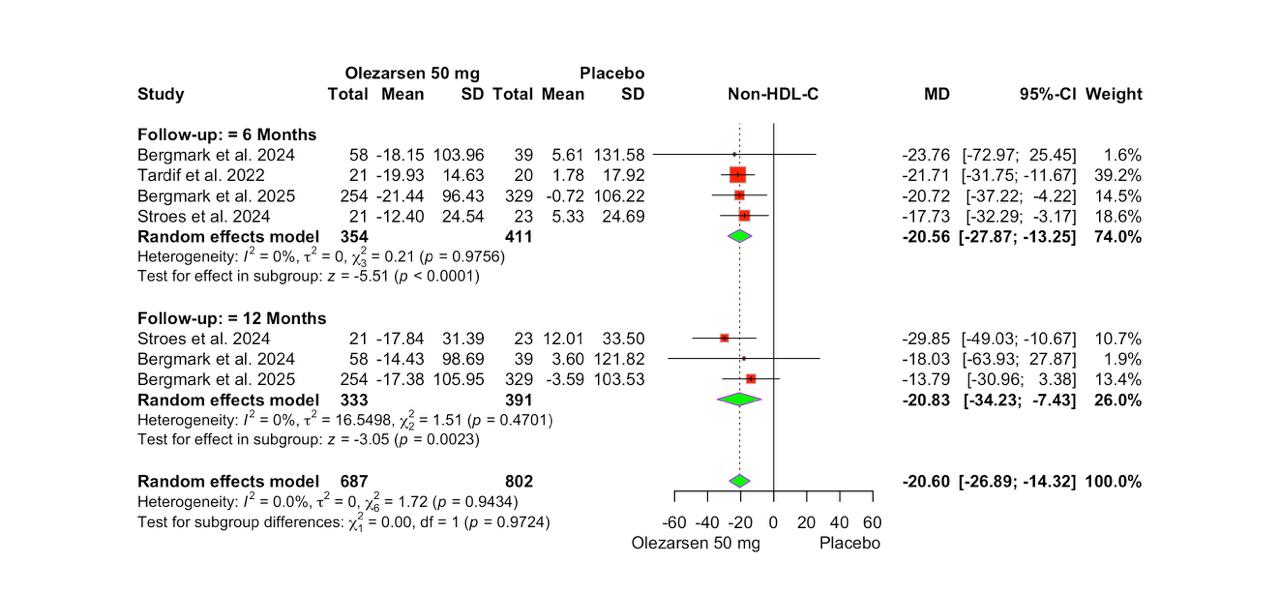


**Supplementary Fig. 5** Forest plot of subgroup analysis by follow-up for changes in Non-HDL-C level for 50 mg dose.


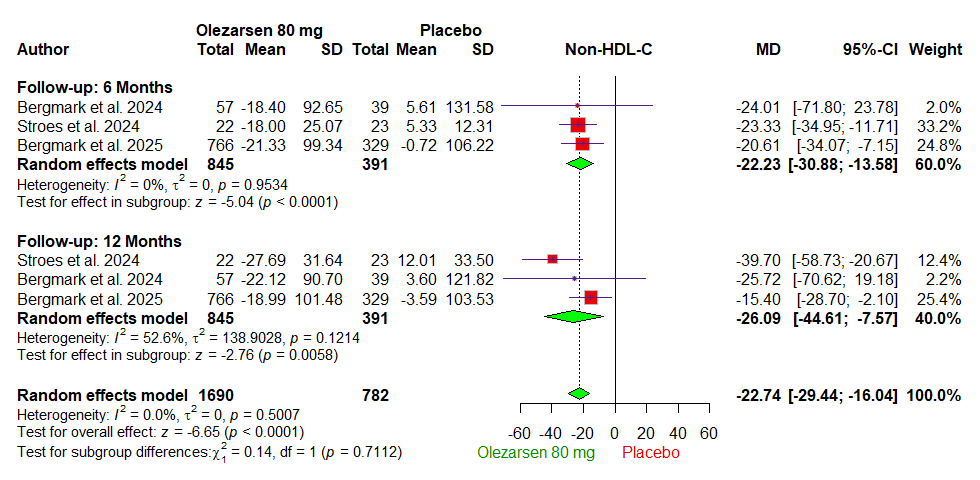


**Supplementary Fig. 6** Forest plot of subgroup analysis by follow-up for changes in Non-HDL-C level for 80 mg dose.


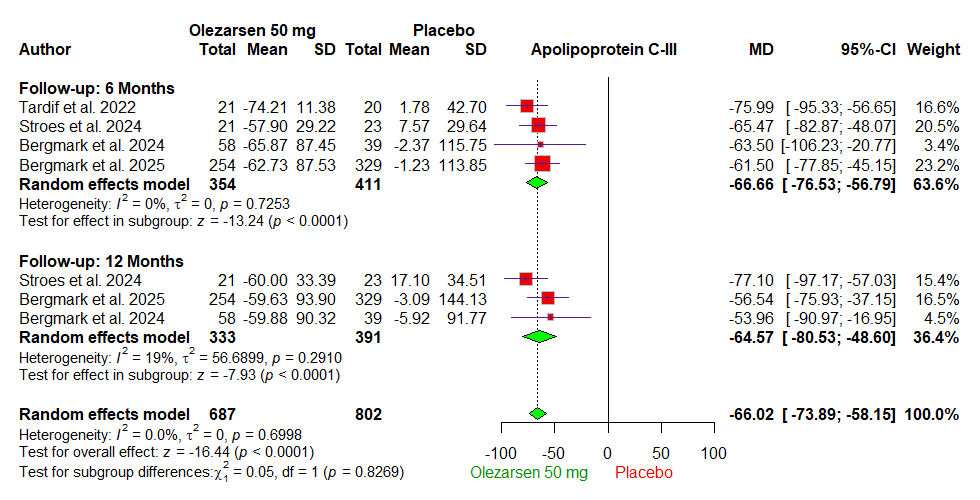


**Supplementary Fig. 7** Forest plot of subgroup analysis by follow-up for changes ApoC-III level for 50 mg dose.


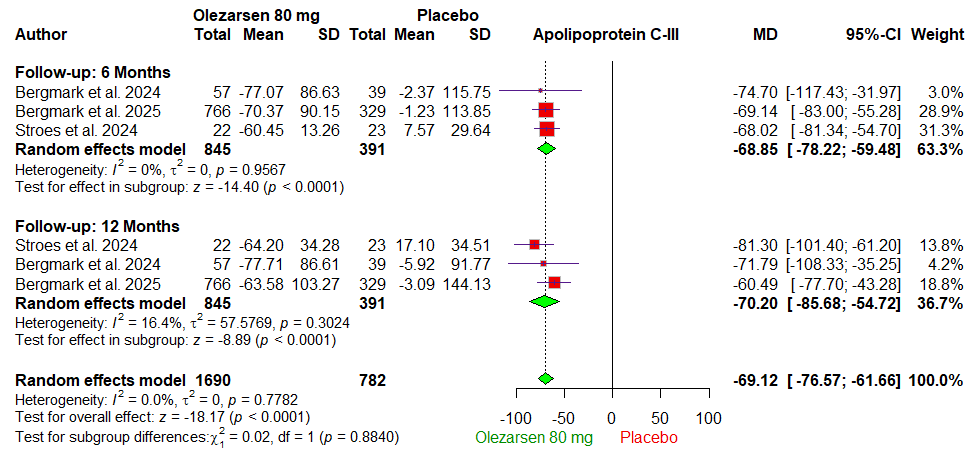


**Supplementary Fig. 8** Forest plot of subgroup analysis by follow-up for changes in ApoC-III level for 80 mg dose.


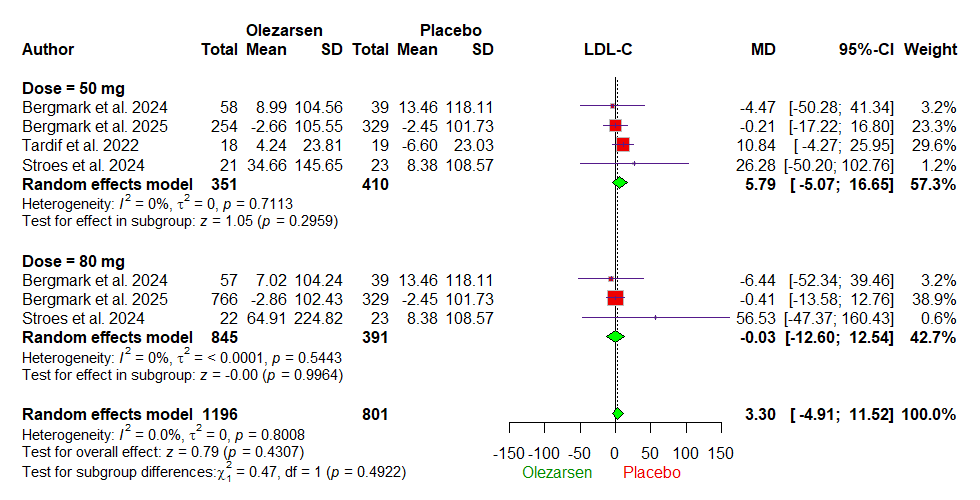


**Supplementary Fig. 9** Forest plot of subgroup analysis by dose for changes in LDL-C level.


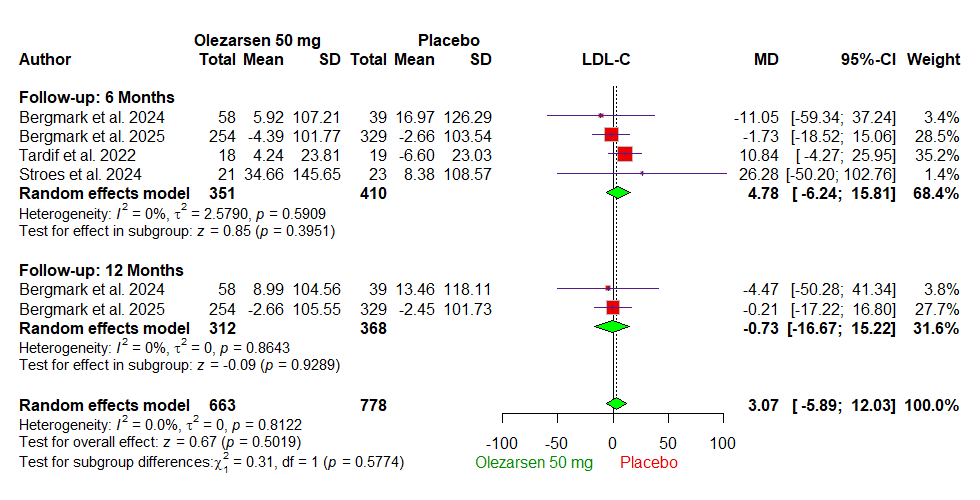


**Supplementary Fig. 10** Forest plot of subgroup analysis by follow-up for changes in LDL-C level for 50 mg dose.


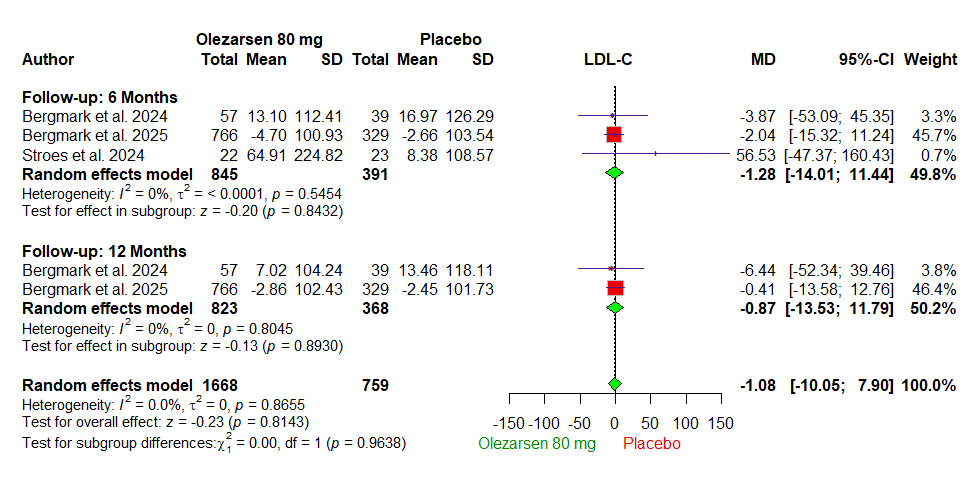


**Supplementary Fig. 11** Forest plot of subgroup analysis by follow-up for changes in LDL-C level for 80 mg dose.


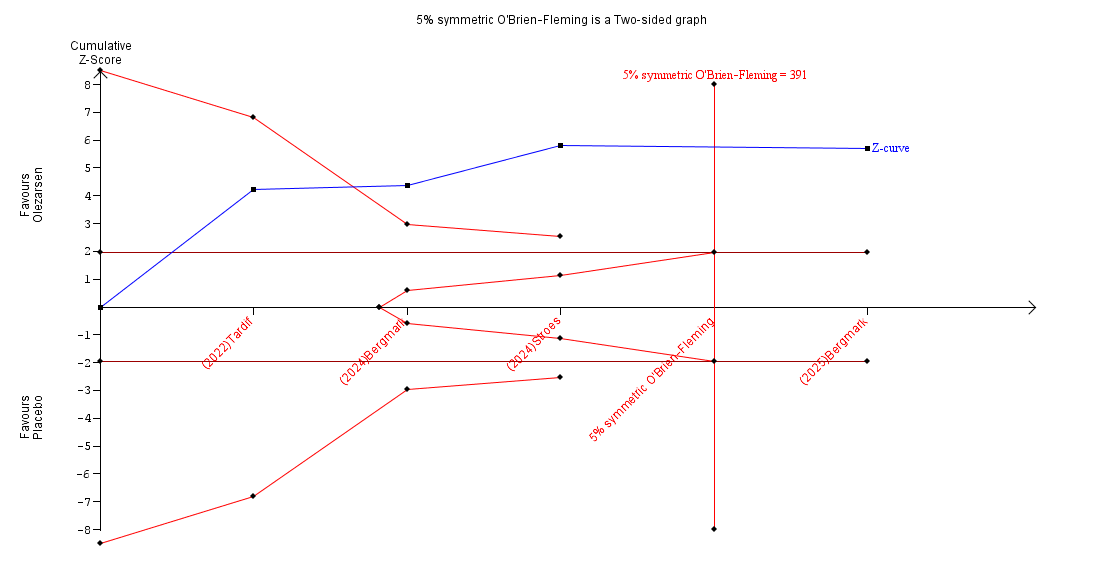


**Supplementary Fig. 12** Trial Sequential Analysis (TSA) of Non-HDL-C change.


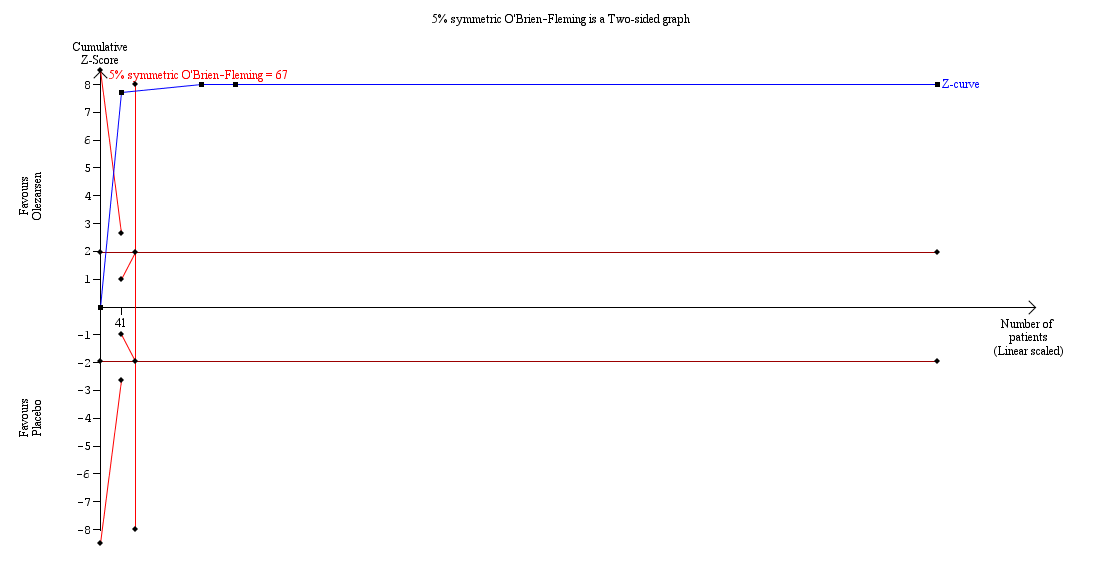


**Supplementary Fig. 13** Trial Sequential Analysis (TSA) of ApoC-III change.


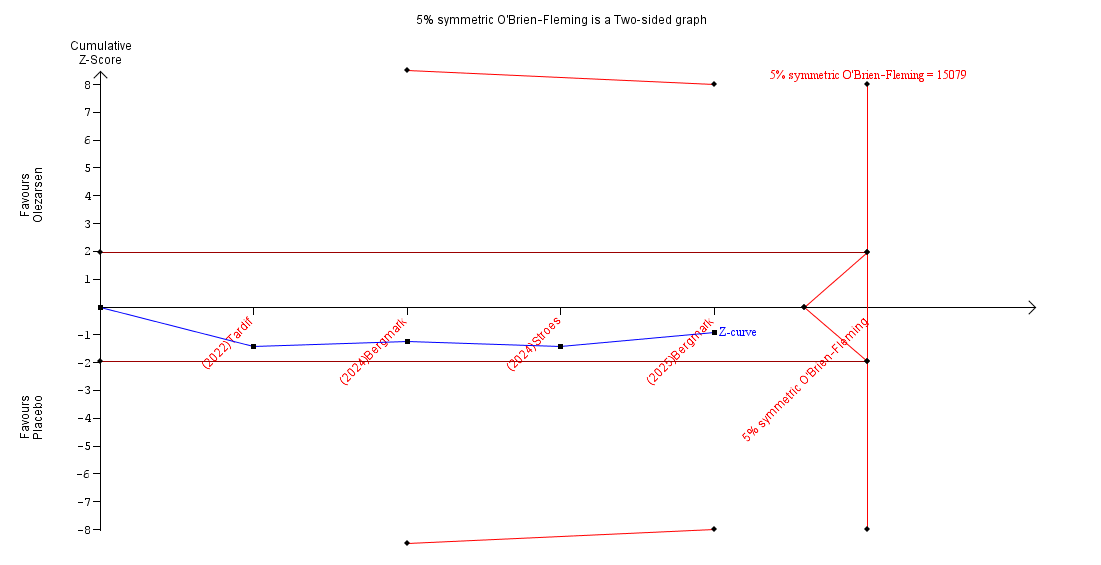


**Supplementary Fig. 14** Trial Sequential Analysis (TSA) of LDL-C change.


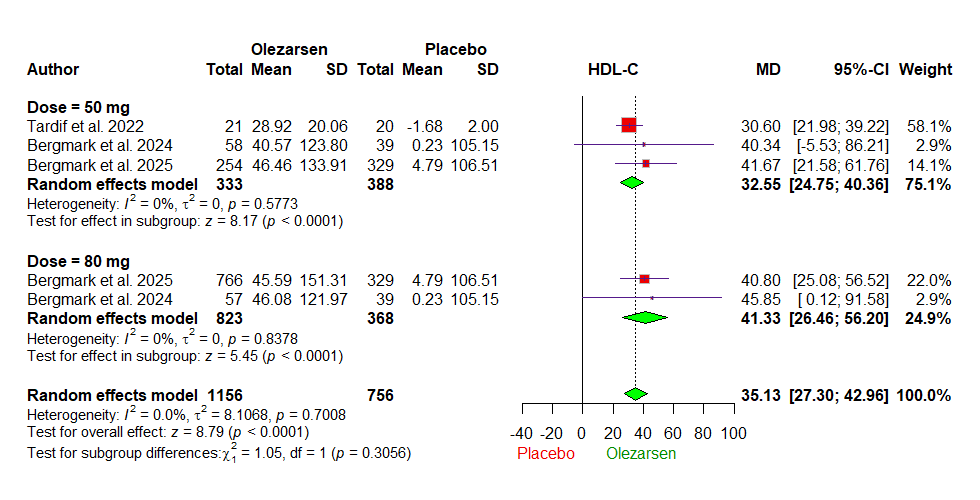


**Supplementary Fig. 15** Forest plot of subgroup analysis by dose for changes in HDL-C level.


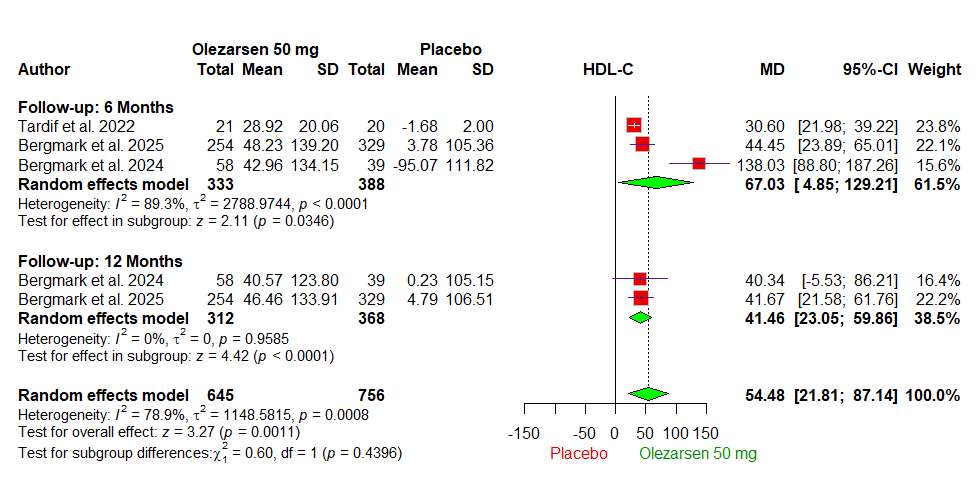


**Supplementary Fig. 16** Forest plot of subgroup analysis by follow-up for changes in HDL-C level in 50 mg dose.


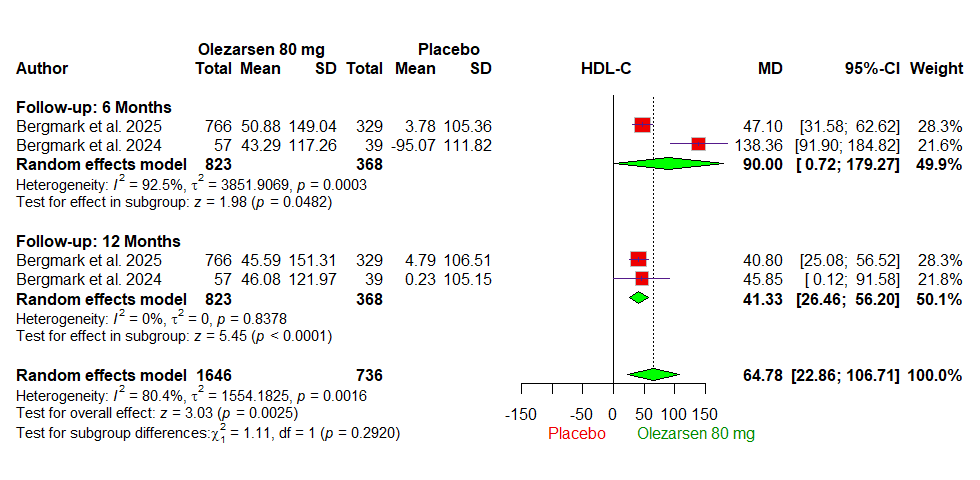


**Supplementary Fig. 17** Forest plot of subgroup analysis by follow-up for changes in HDL-C level in 80 mg dose.


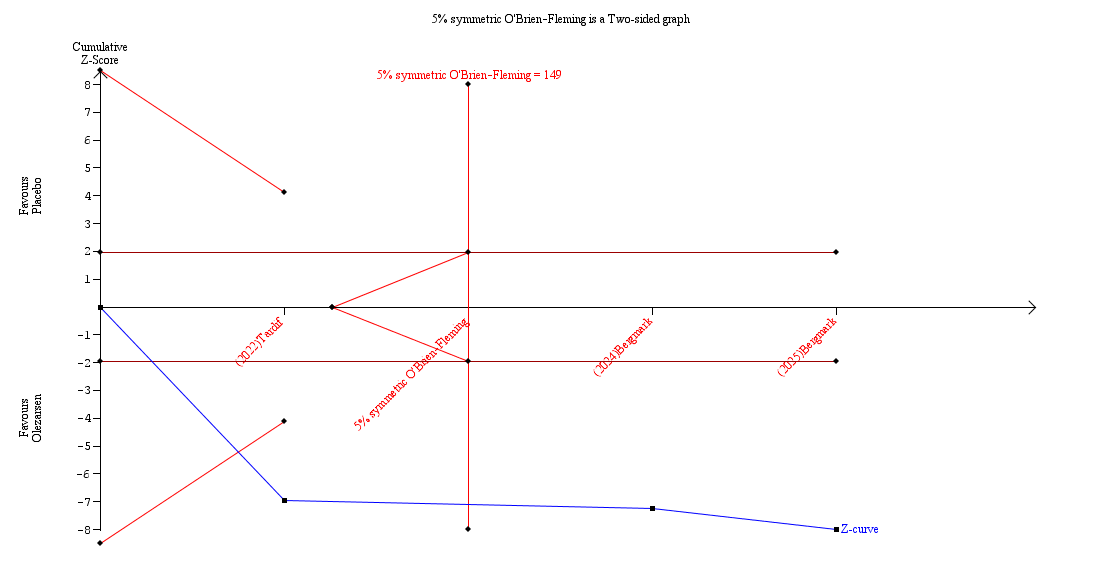


**Supplementary Fig. 18** Trial Sequential Analysis (TSA) of HDL-C change.


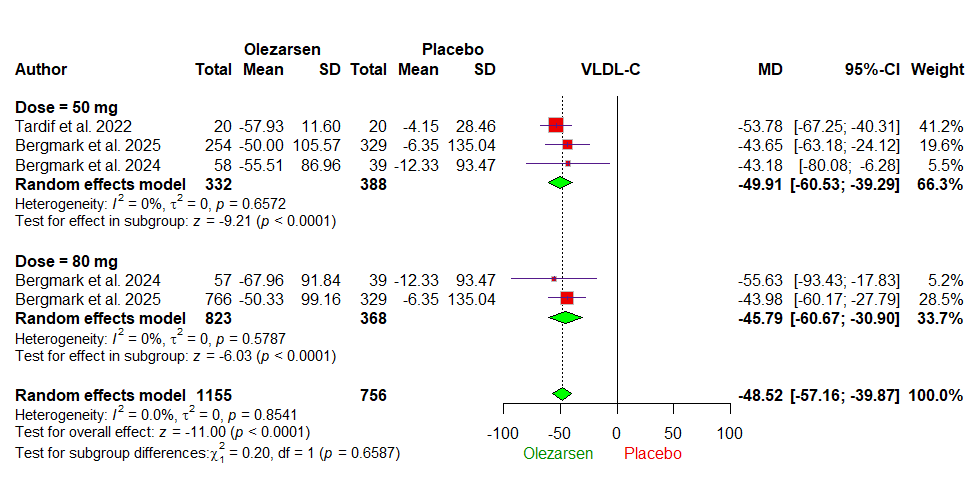


**Supplementary Fig. 19** Forest plot of subgroup analysis by dose for changes in VLDL-C.


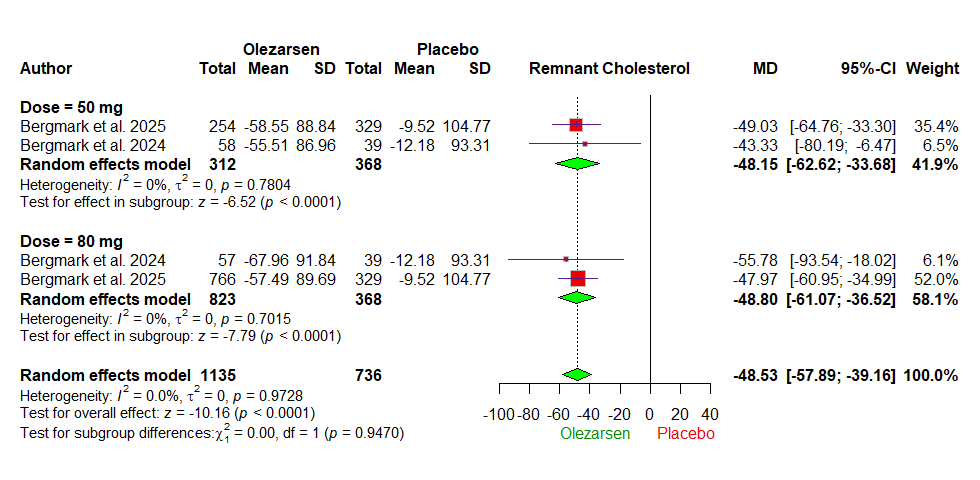


**Supplementary Fig. 20** Forest plot of subgroup analysis by dose for changes in remnant cholesterol.


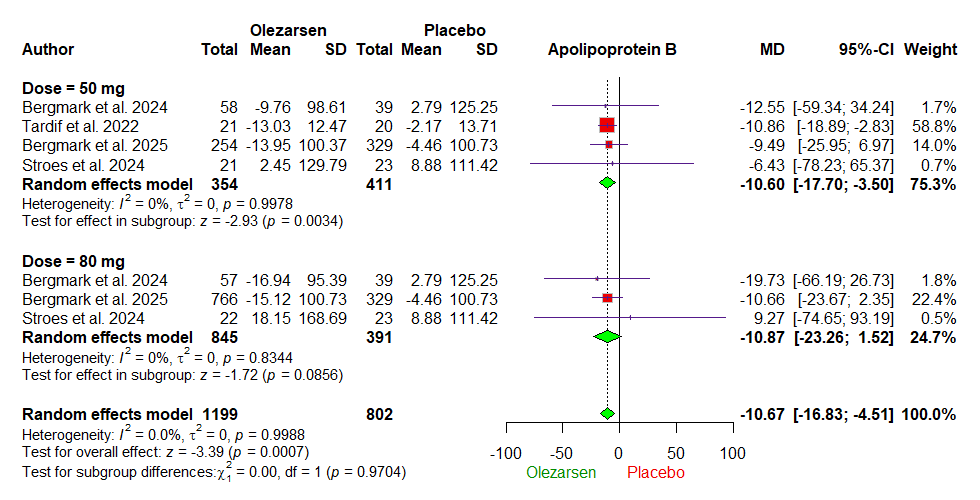


**Supplementary Fig. 21** Forest plot of subgroup analysis by dose for changes in ApoB.


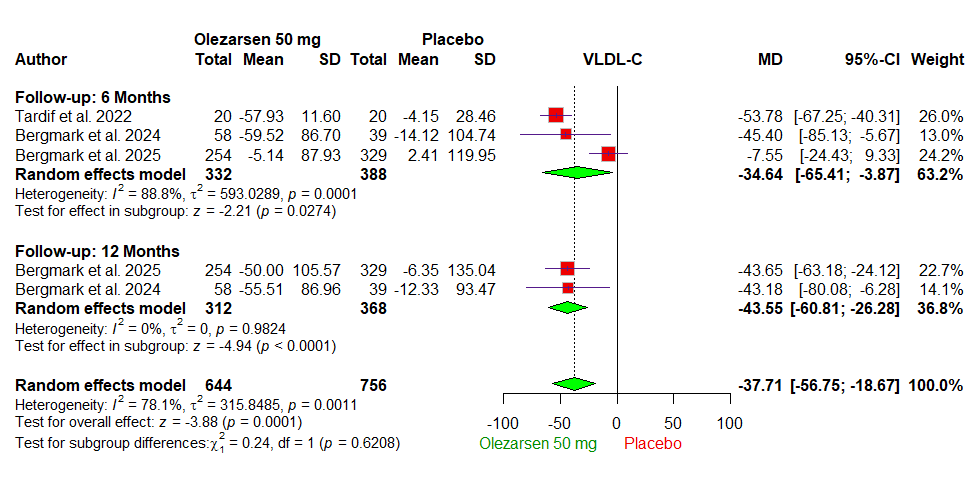


**Supplementary Fig. 22** Forest plot of subgroup analysis by follow-up for changes of VLDL-C in 50 mg dose.


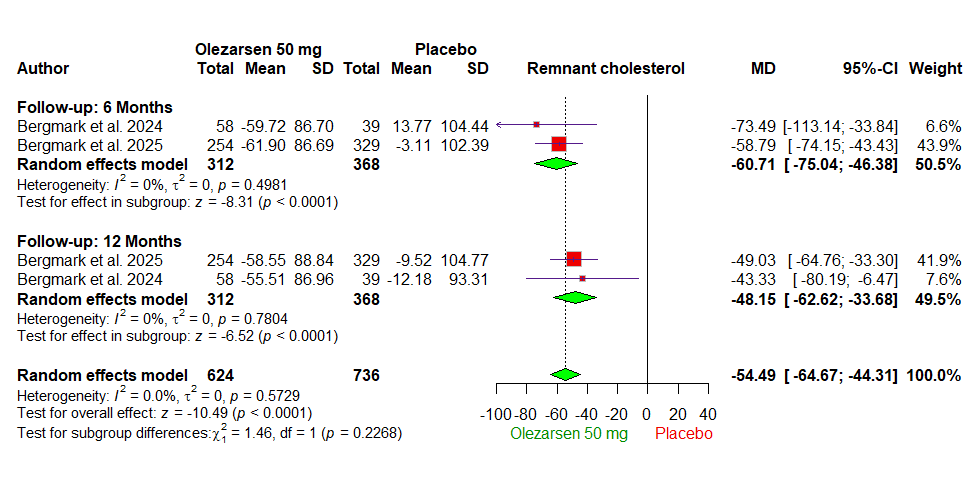


**Supplementary Fig. 23** Forest plot of subgroup analysis by follow-up for changes of remnant cholesterol in 50 mg dose.


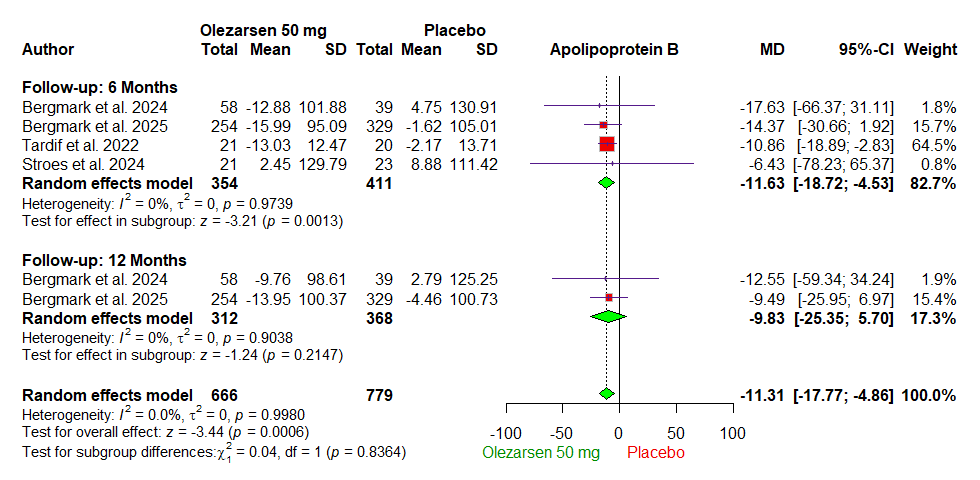


**Supplementary Fig. 24** Forest plot of subgroup analysis by follow-up for changes in ApoB in 50 mg dose.


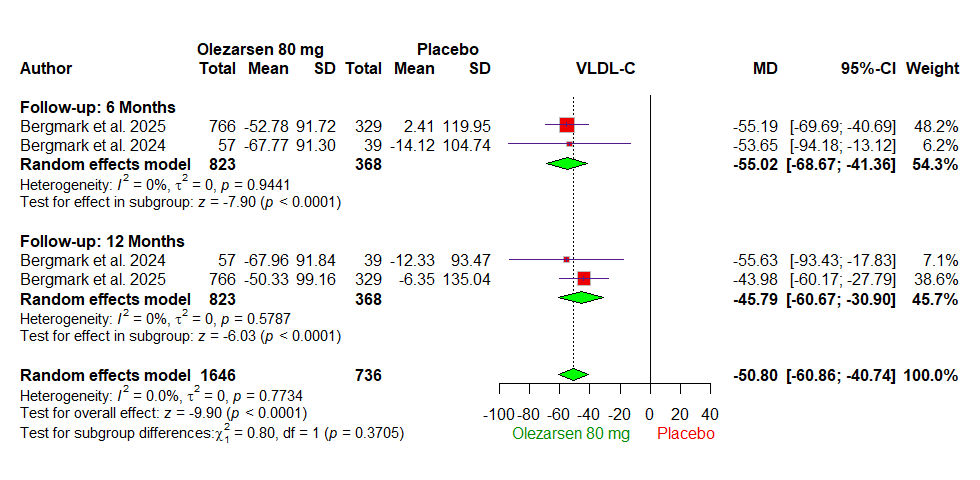


**Supplementary Fig. 25** Forest plot of subgroup analysis by follow-up for changes in VLDL-C in 80 mg dose.


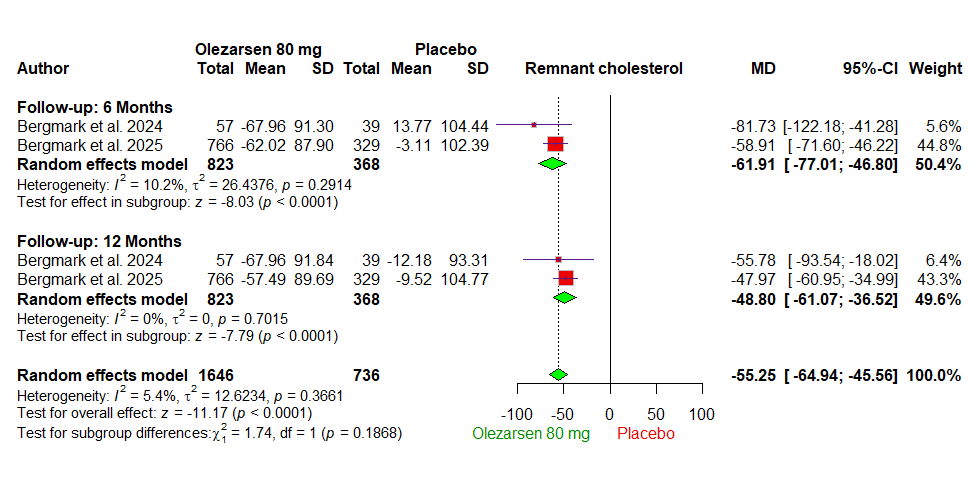


**Supplementary Fig. 26** Forest plot of subgroup analysis by follow-up for changes in remnant cholesterol in 80 mg dose.


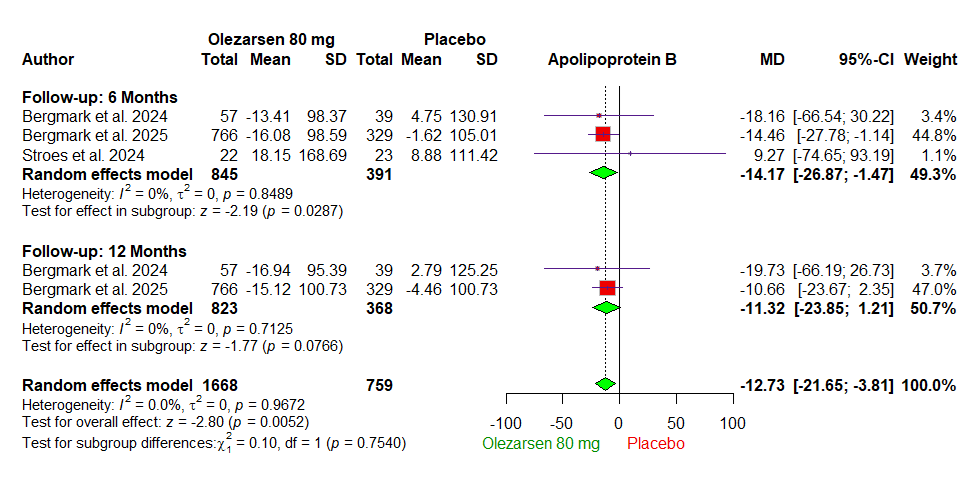


**Supplementary Fig. 27** Forest plot of subgroup analysis by follow-up for changes in ApoB in 80 mg dose.


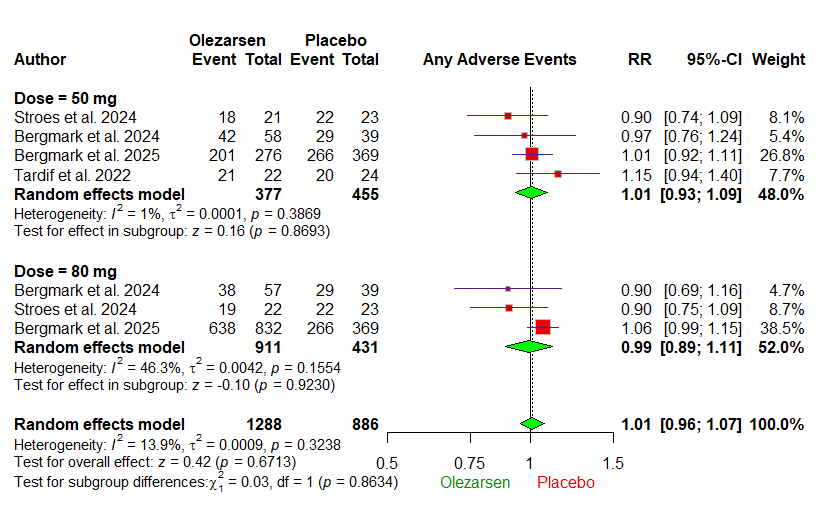


**Supplementary Fig. 28** Forest plot of Any Adverse Events.


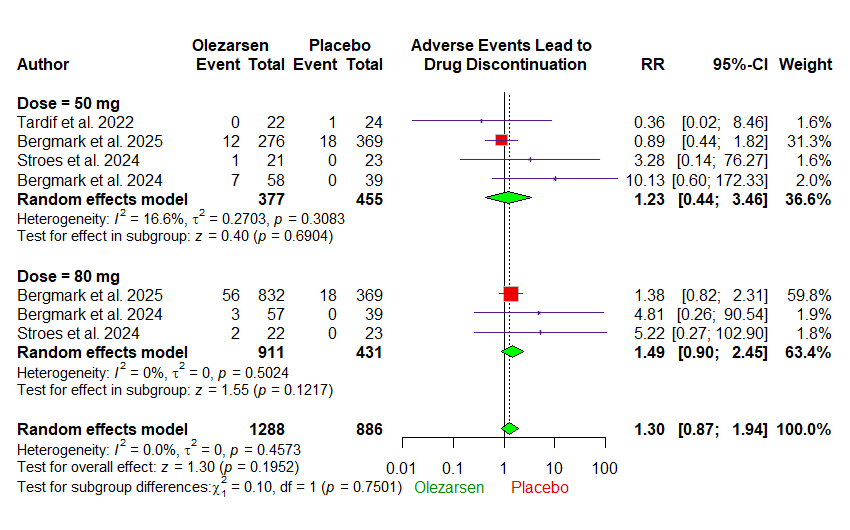


**Supplementary Fig. 29** Forest plot of Adverse Events Leading to Drug Discontinuation.


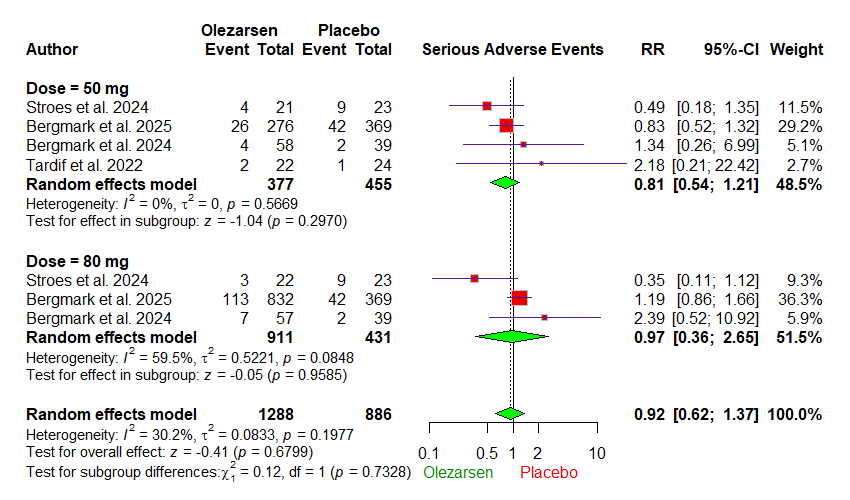


**Supplementary Fig. 30** Forest plot of Serious Adverse Events.


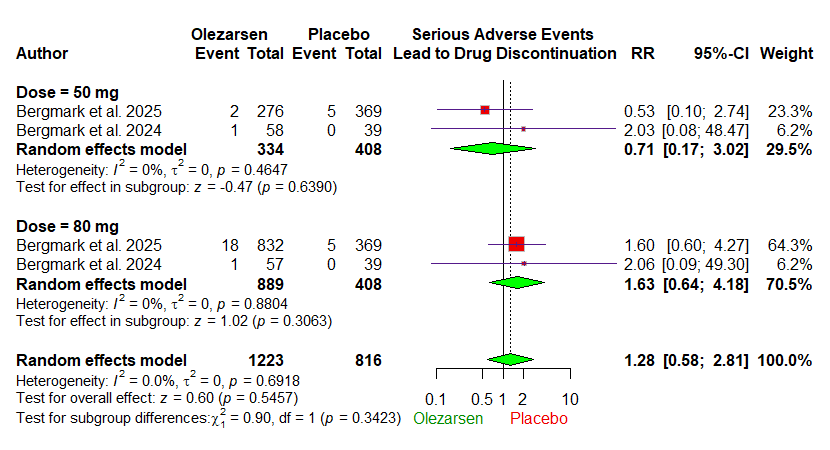


**Supplementary Fig. 31** Forest plot of Serious Adverse Events Leading to Drug Discontinuation.


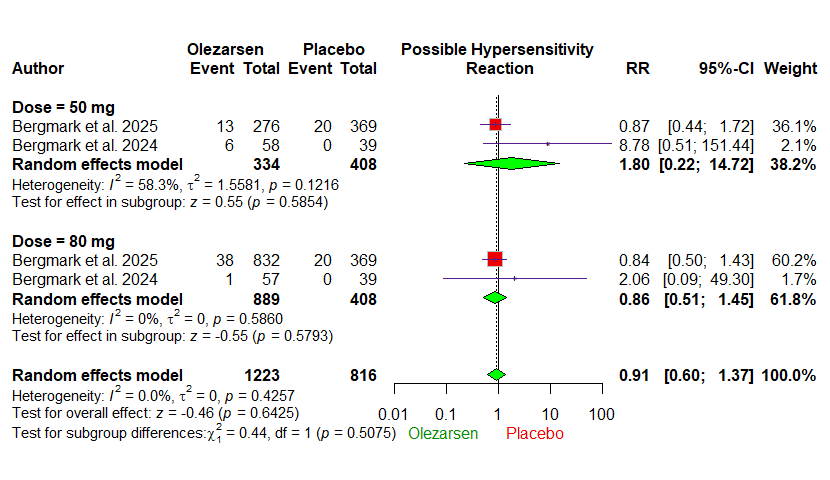


**Supplementary Fig. 32** Forest plot of Possible Hypersensitivity Reaction.


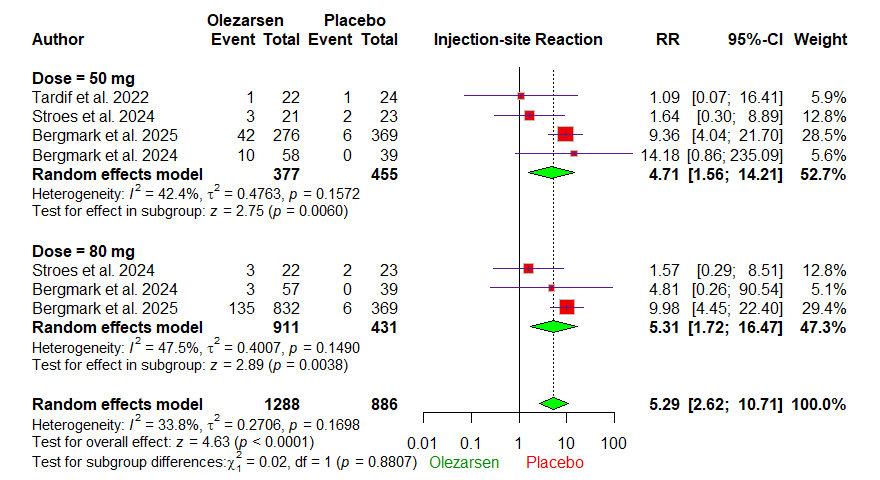


**Supplementary Fig. 33** Forest plot of Injection Site Reaction.


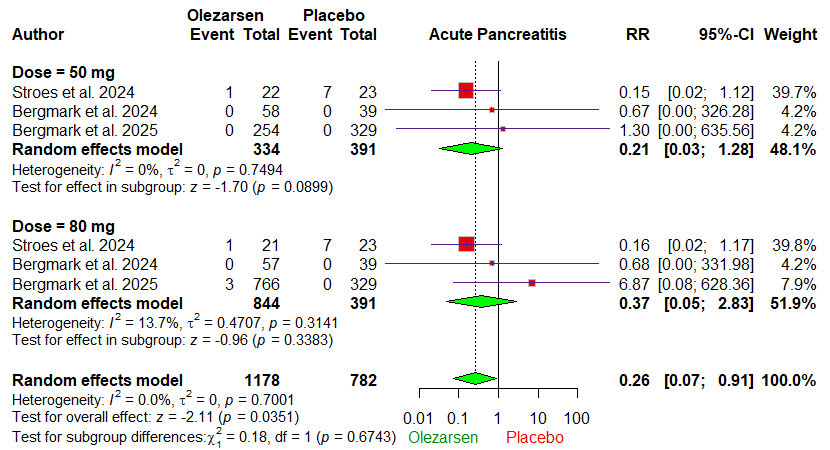


**Supplementary Fig. 34** Forest plot of Acute Pancreatitis.


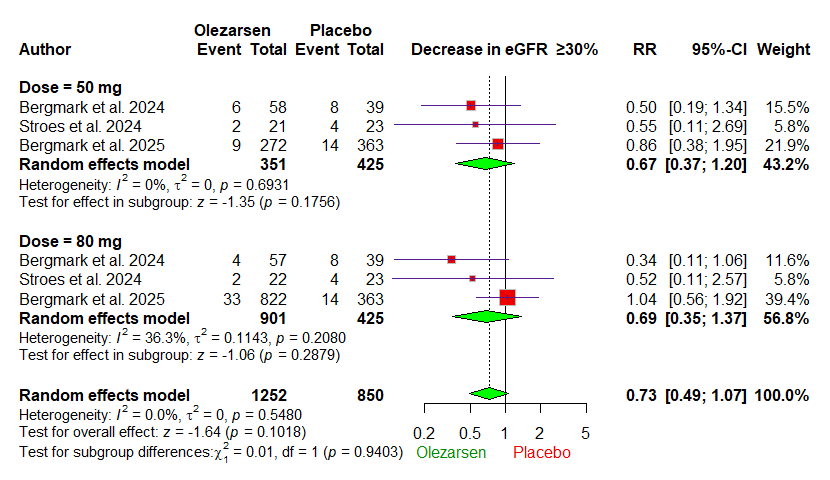


**Supplementary Fig. 35** Forest plot of Decrease in eGFR ≥ 30%.


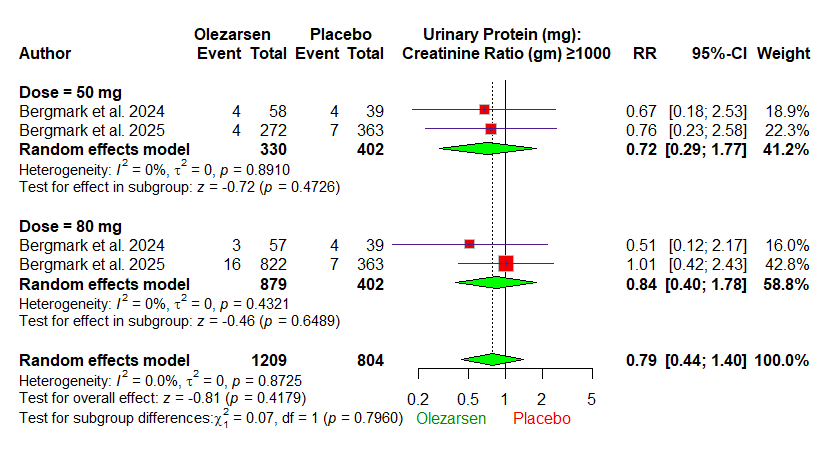


**Supplementary Fig. 36** Forest plot of Urinary Protein: Creatinine Ratio ≥1000.


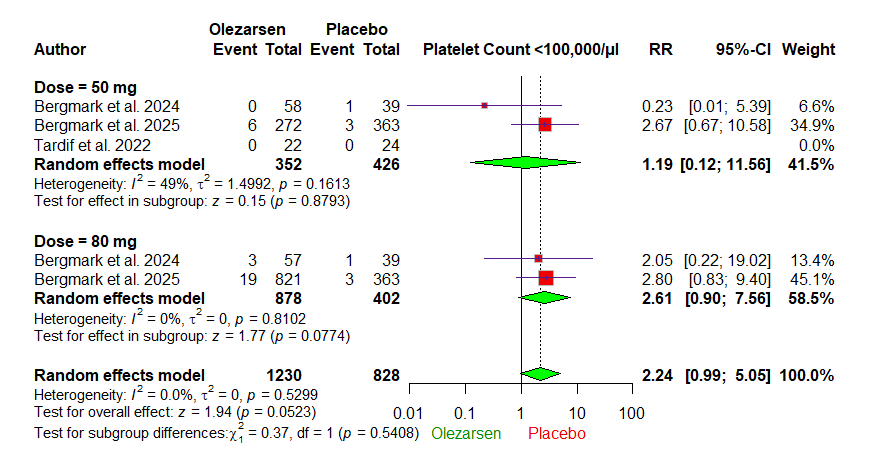


**Supplementary Fig. 37** Forest plot of Platelet Count <100,000/μl.


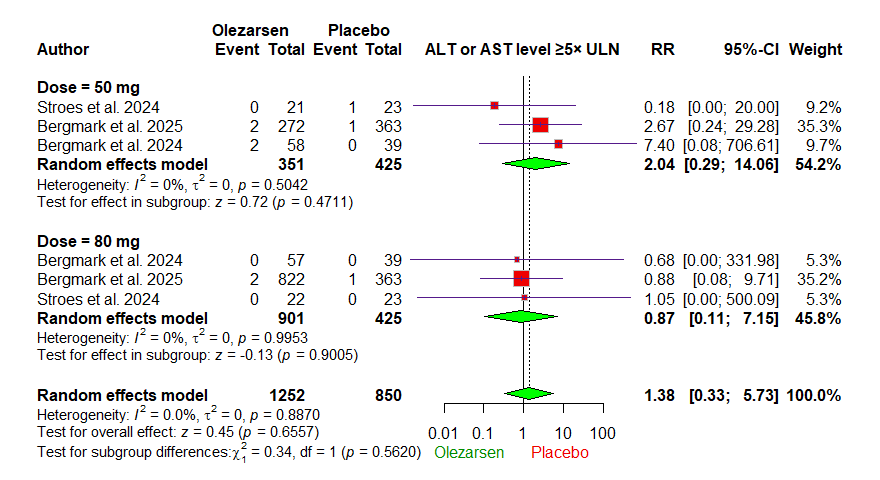


**Supplementary Fig. 38** Forest plot of ALT or AST level ≥5× ULN.


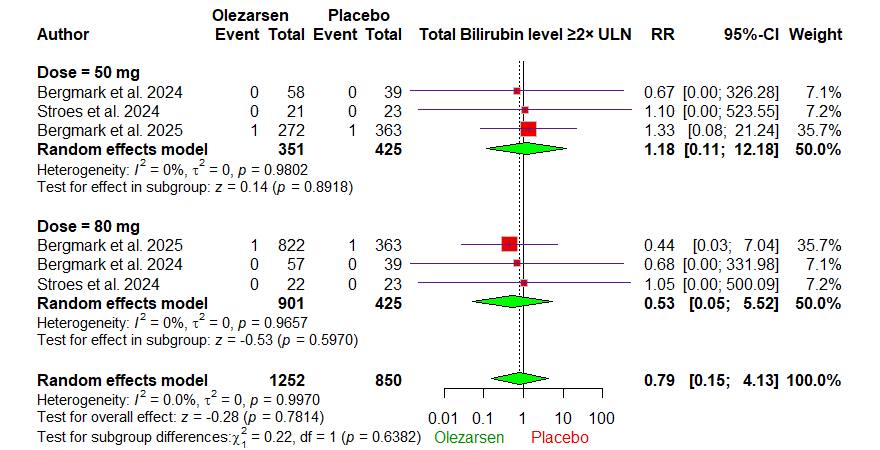


**Supplementary Fig. 39** Forest plot of total bilirubin level ≥2× ULN.


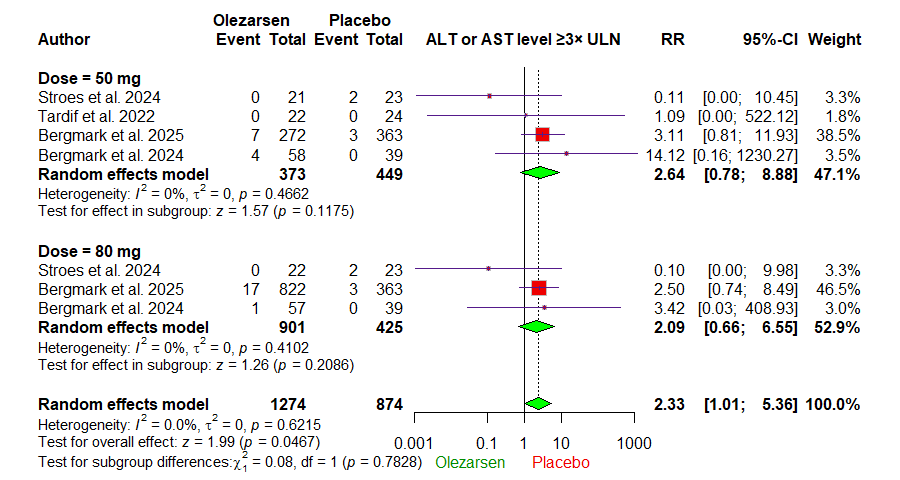


**Supplementary Fig. 40** Forest plot of ALT or AST level ≥3× ULN.


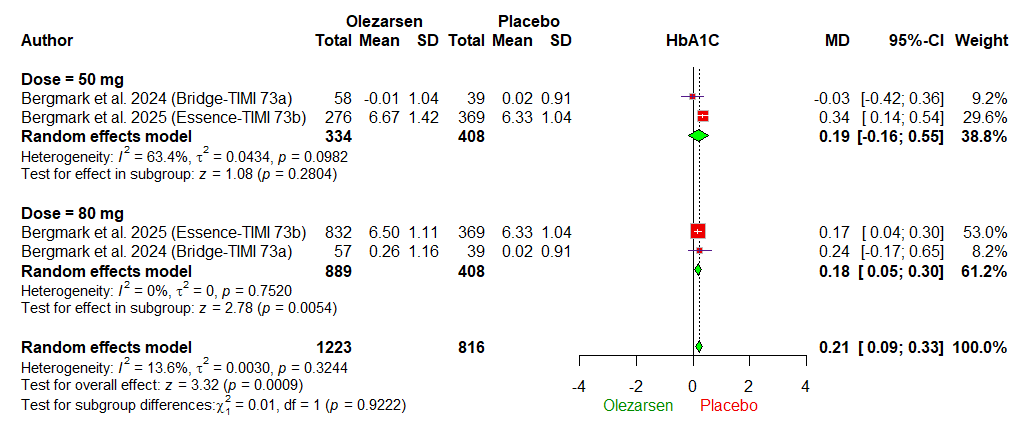


**Supplementary Fig. 41** Forest plot of Change in HBA1C.


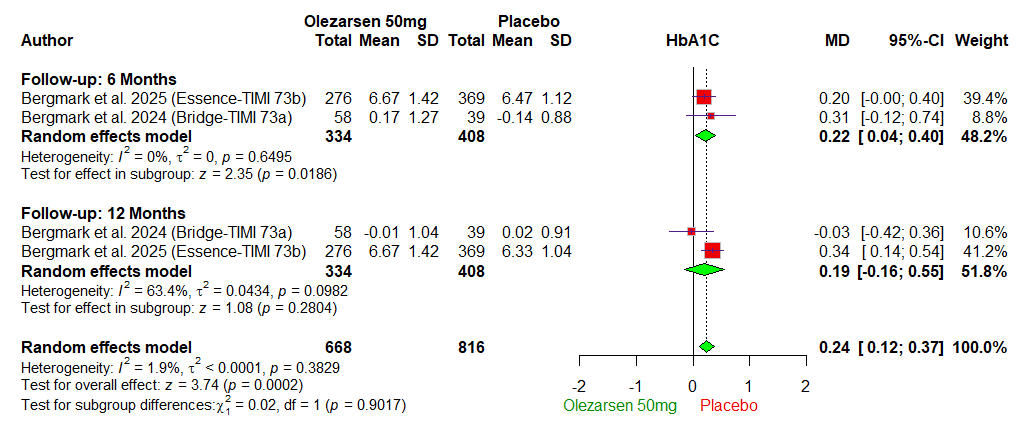


**Supplementary Fig. 42** Forest plot of subgroup analysis by follow-up for HBA1C in 50 mg dose.


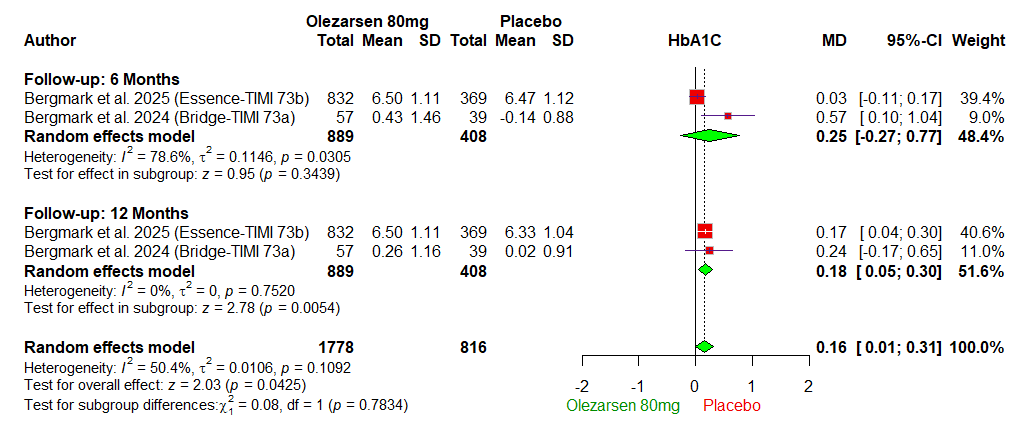


**Supplementary Fig. 43** Forest plot of subgroup analysis by follow-up for HBA1C in 80 mg dose.
